# Polymer-free corticosteroid dimer implants for controlled and sustained drug delivery

**DOI:** 10.1038/s41467-021-23232-7

**Published:** 2021-05-17

**Authors:** Kyle Battiston, Ian Parrag, Matthew Statham, Dimitra Louka, Hans Fischer, Gillian Mackey, Adam Daley, Fan Gu, Emily Baldwin, Bingqing Yang, Ben Muirhead, Emily Anne Hicks, Heather Sheardown, Leonid Kalachev, Christopher Crean, Jeffrey Edelman, J. Paul Santerre, Wendy Naimark

**Affiliations:** 1 Ripple Therapeutics, Toronto, ON Canada; 2 School of Biomedical Engineering, McMaster University, Hamilton, ON Canada; 3 Department of Chemical Engineering, McMaster University, Hamilton, ON Canada; 4 Department of Mathematical Sciences, University of Montana, Missoula, MT USA; 5 Xyzagen Inc., Pittsboro, NC USA; 6 Faculty of Dentistry, University of Toronto, Toronto, ON Canada; 7 Institute of Biomedical Engineering, University of Toronto, Toronto, ON Canada; 8 Translational Biology and Engineering Program, Ted Rogers Centre for Heart Research, Toronto, ON Canada

**Keywords:** Drug delivery, Medicinal chemistry, Biomedical materials

## Abstract

Polymeric drug carriers are widely used for providing temporal and/or spatial control of drug delivery, with corticosteroids being one class of drugs that have benefitted from their use for the treatment of inflammatory-mediated conditions. However, these polymer-based systems often have limited drug-loading capacity, suboptimal release kinetics, and/or promote adverse inflammatory responses. This manuscript investigates and describes a strategy for achieving controlled delivery of corticosteroids, based on a discovery that low molecular weight corticosteroid dimers can be processed into drug delivery implant materials using a broad range of established fabrication methods, without the use of polymers or excipients. These implants undergo surface erosion, achieving tightly controlled and reproducible drug release kinetics in vitro. As an example, when used as ocular implants in rats, a dexamethasone dimer implant is shown to effectively inhibit inflammation induced by lipopolysaccharide. In a rabbit model, dexamethasone dimer intravitreal implants demonstrate predictable pharmacokinetics and significantly extend drug release duration and efficacy (>6 months) compared to a leading commercial polymeric dexamethasone-releasing implant.

## Abstract (web summary)

Polymer-based systems are often considered a necessity for controlled drug delivery, but have well-known limitations. Here, the authors report on drug delivery implants formed solely from corticosteroid dimers, which demonstrate controlled release and overcome many of the challenges of polymer-based systems.

## Introduction

Beginning in the 1960’s, biostable and biodegradable polymers were introduced to formulate drugs into implants with controlled release profiles. Today, polymeric drug carriers are used extensively in pharmaceutical and medical device applications to provide local or systemic controlled, tunable drug delivery^1–4^. By modulating spatiotemporal distribution of drugs in the body, these systems minimize systemic side effects, reduce drug loading requirements, and more efficiently target tissue sites. Biodegradable polymers are particularly attractive since they fully resorb in the body and provide flexibility in modulating release kinetics and functional properties based on the polymer backbone (e.g., ratio of block co-polymers^5–7^).

Polymers such as polycaprolactone and poly(lactic-co-glycolic acid) (PLGA) have long been held as the gold standard polymer carrier systems for controlled drug delivery applications. Corticosteroids, which have utility in the treatment of numerous inflammatory conditions (e.g., ocular conditions^8^, osteoarthritis^9^, asthma^10^, and others^11^), are one drug class that has benefited from the use of polymeric delivery systems, as side effects associated with their systemic use (e.g., cortisol suppression and cataracts, amongst others^12,13^) can be minimized by local, controlled release^7,9,14–16^. Despite the advantages of these products, they are subject to limited drug loading capacity (e.g., typical systems are made up of 40–90% polymer carrier), burst release profiles, and sub-optimal release kinetics^3,5,16,17^, resulting in excessive drug levels leading to both local and systemic side effects, and sub-optimal dose durations^1,9,16,18^. Unpredictable release kinetics can be attributed in part to the bulk erosion mechanism of polymer degradation. For example, PLGA implants are permeated by water, hydrolyzing the polymer matrix, and forming pores for drug diffusion^19,20^. Acidic degradation by-products generated from polymers such as PLGA (e.g., lactic acid, glycolic acid) have been reported to cause heightened inflammation^21,22^. Drug-free polymer remnants can remain following complete drug delivery and have been associated with local tolerability and toxicity^22–25^. In contrast, an ideal drug delivery system, as outlined by Langer and Peppas in 1981, would release drug through a surface-mediated process; be completely eliminated with non-harmful degradation products; would not induce adverse tissue reactions; and would have degradation rates that are predictable and can be altered by simple chemical changes^5^.

In this study, we report on the use of low molecular weight corticosteroid dimers that possess many qualities required of an ideal drug delivery system. Corticosteroid dimers are used as building blocks for the fabrication of material constructs that do not depend on polymeric carriers. They are prepared through well-defined and scalable chemical synthesis processes and formed into standalone implants with a number of standard polymer processing techniques. The implants are composed entirely of the dimer and have the capacity to provide targeted and controlled drug delivery, undergoing surface erosion to yield highly predictable sustained release kinetics. The elimination of a polymer matrix results in high drug loading, enabling implants to be made significantly smaller when compared to conventional polymeric systems. Due to the surface erosion mechanism of release and the flexibility in molecular design and implant form, drug doses can be precisely tuned to achieve a target therapeutic window. The use of an implant formed solely from a dexamethasone dimer is used to demonstrate the ability to provide sustained, controlled intraocular release of the anti-inflammatory corticosteroid dexamethasone in vivo to counteract an acute lipopolysaccharide-mediated inflammatory response in rats as well as vascular endothelial growth factor-induced vascular leakage in a chronic rabbit model.

## Results and discussion

### Processing polymer-free corticosteroid dimers into engineered forms

The use of a drug dimer to form stand-alone implants and provide sustained drug release was initially demonstrated with dexamethasone (Dex). Dex has wide-ranging applications and can be delivered systemically or more directly at the desired site of action (e.g., ophthalmic conditions, osteoarthritis, respiratory diseases, amongst others^1,8,10,11,26^). In both instances, however, the lack of a controlled delivery system for controlling drug levels and duration of drug exposure, as well as the use of drug delivery systems with uncontrolled burst release profiles, requires excessive dosing that is associated with systemic side effects, ranging from psychiatric (e.g., anxiety, depression) to endocrine (e.g., cortisol suppression) and cardiovascular (e.g., cardiomyopathy) in nature, in addition to others^26^. As a result, Dex would benefit from an improved sustained-release approach to conventional polymeric systems.

**Fig. 1:**
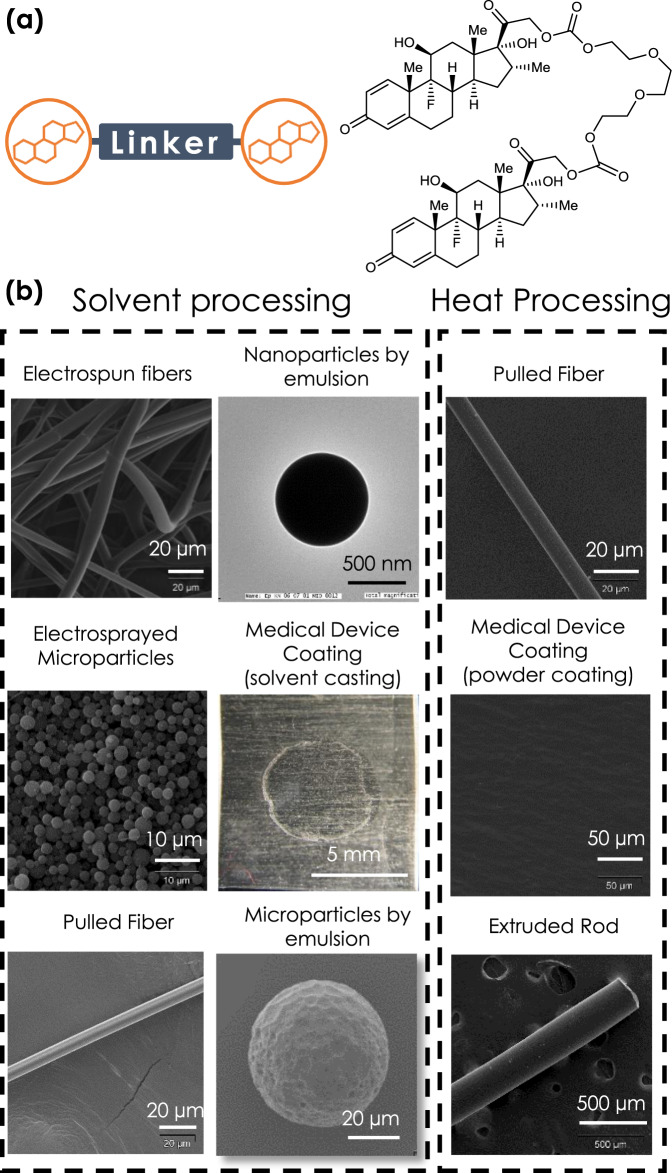
Processing of drug dimers into stand-alone drug delivery vehicles. **a** Schematic demonstrating the general form of the dimers, and the chemical structure of a representative dimer molecule made with the corticosteroid dexamethasone using a triethylene glycol (TEG) linker. (**b** Transmission electron microscopy (nanoparticles), light micrographs (coatings), and scanning electron microscopy (SEM) (all others) images demonstrating steroid dimers processed into different physical forms through thermal processing (fibers, coatings, extruded rods) and solvent processing (nanoparticles, microparticles, fibers, coatings, fibrous meshes).

A Dex dimer was synthesized through a one-step synthesis, using a triethylene gycol (TEG) linker and two Dex molecules (Dex-TEG-Dex) (Fig. 1a, Supplementary Figure 1). Structures were confirmed by nuclear magnetic resonance spectroscopy (NMR) (Supplementary Fig. 1) and mass spectrometry (*m*/*z*: [M + H]+ Calculated for C52H69F2O16 987.4554; Found 987.4549). Due to its simple design and the ability to synthesize the dimers as monodisperse entities, Dex-TEG-Dex has well-defined degradation products (free Dex, Dex-TEG, TEG, CO_2_). The melting point (*T*_m_) of Dex-TEG-Dex was 167 °C, in comparison to a *T*_m_ of 262 °C for Dex^27^. While the synthesis of drug conjugates has been reported in the literature, this synthetic approach is typically pursued for the purpose of changing drug solubility, biodistribution, or increasing half-life when administered as solubilized compounds^28^. In contrast, the utility of the disclosed dimers is their ability to be fabricated into stand-alone implants. When Dex-TEG-Dex is processed using established techniques, it yields amorphous engineered shapes and forms. Processability of Dex-TEG-Dex was demonstrated using standard heat^29,30^ and solvent^30–32^ polymer processing methods. Importantly, no additives, polymers, excipients, or other agents, routinely required to physically stabilize drugs into a formed implant, were needed in the manufacturing process^30–33^. Heat-based processing was used to form fibers (fiber pulling), device coatings (powder coating), and rods (melt extrusion) (Fig. 1b). Common solvent-based approaches were used to successfully generate polymer and excipient free drug dimer materials, including fibrous meshes (electrospinning), microparticles (electrospraying, emulsion), nanoparticles (emulsion, Supplementary Fig. 2), and medical device coatings (spray coating, dip coating, solvent casting) (Fig. 1b).

### Characterization of materials formed solely from a processed Dex dimer

**Fig. 2:**
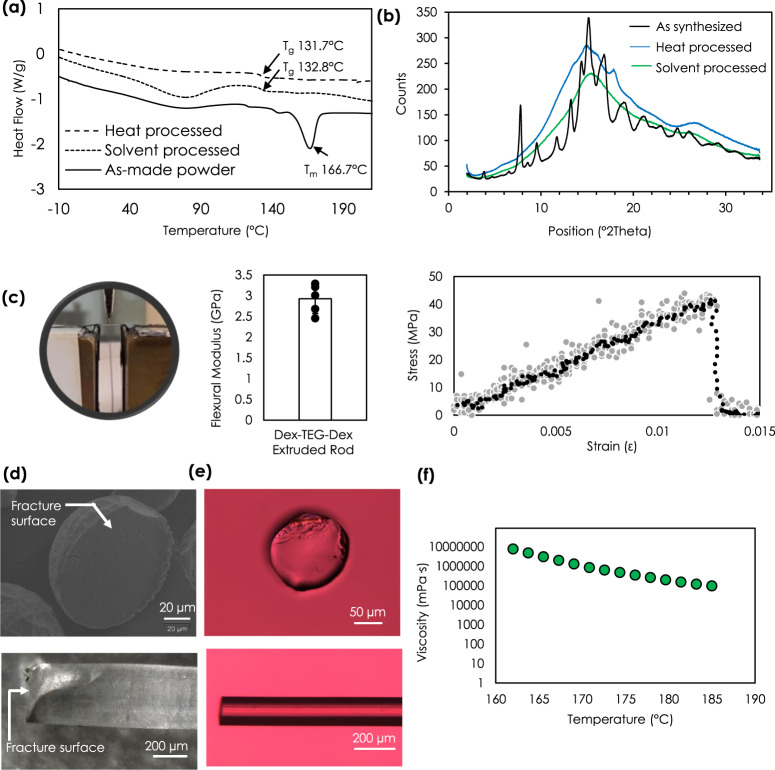
Physical characterization of drug dimers and their processed forms. **a** Differential scanning calorimetry thermograms for crystalline dimers compared to solvent (fibrous mesh) and thermal (pellet) processed forms, demonstrating a transition from a crystalline to amorphous state. *T*_g_ – glass transition temperature. *T*_m_ – melting temperature. **b** Powder X-ray diffraction diffractogram demonstrating the crystalline nature of the synthesized dimer relative to the amorphous structure of the processed forms. **c** Experimental set-up, representative stress-strain curve, and flexural modulus for 3-point bend testing of an extruded rod of dexamethasone (Dex) dimer (Dex-TEG-Dex). Gray circles represent the raw data and black circles represent the 10-point moving average. *N* = 5. Data represent the mean ± standard deviation. **d** SEM micrograph of a fractured microparticle and light micrograph of an extruded rod following 3-point bend testing. **e** Polarized light microscopy of a longitudinal view and cross-section of an extruded rod, demonstrating the absence of crystalline structure. **f** Representative curve demonstrating rheometric characterization of the heat-processed Dex-TEG-Dex.

The physical nature and stability of Dex-TEG-Dex materials can be defined by fundamental material properties. Differential scanning calorimetry (DSC) analysis indicated a glass transition temperature (*T*_g_) of 131-133 °C, and the absence of a classical T_m_, when the materials were prepared by melt or solvent processing. This suggests a transition to an amorphous state from that of the original crystalline as-made dimer (Fig. 2a). The amorphous nature of the processed form was confirmed by X-ray diffraction (Fig. 2b, Supplementary Fig. 3). The viscosity of the Dex-TEG-Dex material decreased in a temperature-dependent manner (Fig. 2f), an important feature for tailoring thermal processing approaches for fabricating these dimer materials (e.g., melt extrusion). When the extruded rods were subjected to a 3-point bend test, they were determined to have a flexural modulus value of 2.92 ± 0.35 GPa, which is comparable to rigid plastics such as polystyrene^34^ (Fig. 2c). Analysis of the fracture surface post-3-point bend testing indicated a fracture pattern typical for glassy materials (Fig. 2d)^35^. Imaging of the fracture surface for the extruded rods (light microscopy, Fig. 2d, polarized light microscopy, Fig. 2e) and microparticles (scanning electron microscopy (SEM), Fig. 2d) demonstrate the complete absence of crystalline features. These images further highlight the unique physical nature of the formed implant generated with the processed dimer, demonstrating physical characteristics necessary for producing solid implants and maintaining their form, as well as their potential use as drug delivery vehicles without the need for a polymer carrier or other binding agent.

Amorphous drugs, representing a higher energy state than their crystalline counterparts, are typically associated with higher solubility^36^. In many cases, amorphous drugs are pursued specifically for this purpose, with the goal of maximizing solubility of hydrophobic drugs that would otherwise not possess suitable solubility for their intended use^37^. It was thus important to demonstrate that Dex-TEG-Dex that had been processed into the amorphous state did not undergo crystallization and remained in the amorphous form following storage. If crystallization should occur it could have the potential to impact dissolution and thus drug release kinetics. Following exposure to shelf-life storage conditions, extruded Dex-TEG-Dex implants were evaluated by polarized light microscopy and showed no evidence of crystallinity within the amorphous implants (Supplementary Fig. 4).

### Dex dimer implants undergo surface erosion to release free drug

**Fig. 3:**
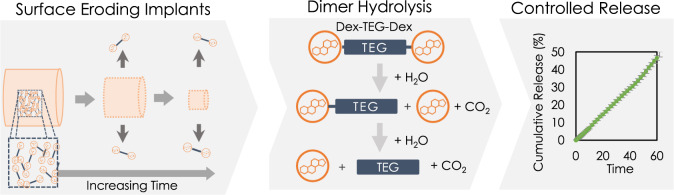
Mechanism of drug release from a drug dimer material. Schematic illustrating the proposed mechanism for the release of free drug from a dimer implant.

**Fig. 4:**
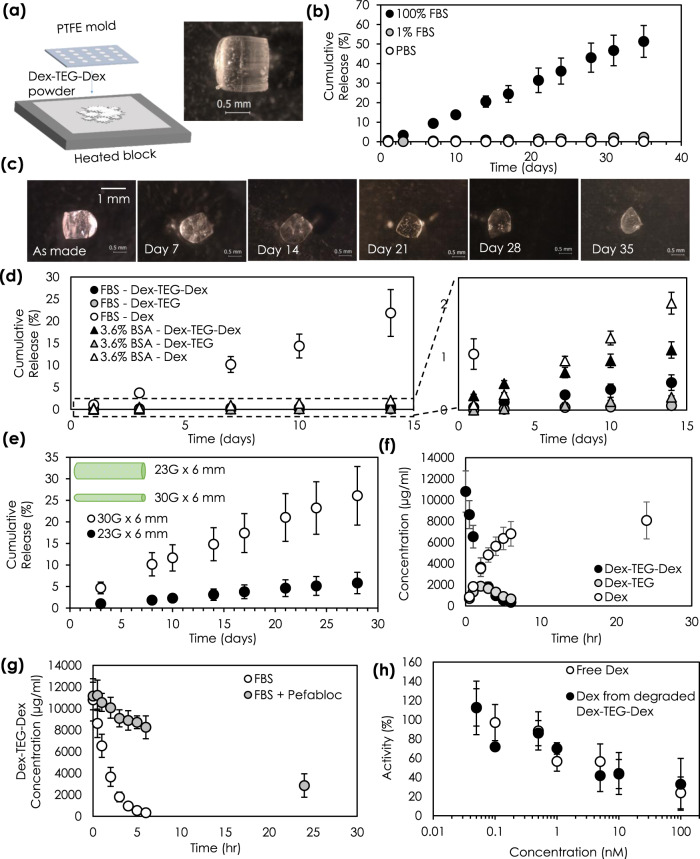
Characterization of the drug release behavior of a corticosteroid dimer implant. **a** Method of thermally processing crystalline dimers into pellets for in vitro release testing. **b** Release kinetics of dexamethasone from thermally processed pellets in 100% fetal bovine serum (FBS), 1% FBS in phosphate-buffered saline (PBS) (pH 7.4), and PBS (pH 7.4) (2 ml). Total drug loading was 1.17 ± 0.24 mg, 1.13 ± 0.17 mg, and 0.85 ± 0.03 mg dexamethasone (Dex) for 100% FBS, 1% FBS, and PBS release conditions, respectively. *N* = 3 samples. **c** Light micrographs demonstrating the surface erosion phenomenon, where pellets in FBS decrease in size over time as dimer is solubilized from the surface and degraded into free drug, resulting in surface erosion-based release kinetics. **d** Comparison of free dexamethasone release profiles in equivalent protein environments with differing hydrolytic enzyme activity (FBS (high) and 3.6% bovine serum albumin (BSA) (low)) in PBS pH 7.4 (2 ml). Total drug loading was 1.28 ± 0.19 mg Dex. N=3 samples for FBS and N=6 samples for BSA. **e** Demonstration of release rate and its dependence on sample diameter. Total drug loading of 63 ± 16 µg for 30 G × 6 mm implants and 348 ± 141 µg for 23 G x 6 mm implants. *N* = 6 samples for 23 G and *N* = 5 samples for 30 G implants. Due to the surface erosion mechanism of release, material diameter is directly correlated to duration of release. Degradation kinetics of solubilized dimer in **f** fetal bovine serum (N=3 samples) showing full conversion to free drug (**g**) with and without the presence of a protease inhibitor (Pefabloc, 1 mg/ml) (*N* = 3 samples). **h** Inhibition of LPS-induced stimulation of PGE2 production by primary human monocytes in the presence of free dexamethasone and dexamethasone released from Dex-TEG-Dex (N=3). In all panels, data represent the mean ± standard deviation.

Next, we sought to understand the ability of processed Dex-TEG-Dex implants to provide controlled release of Dex. Figure 3 illustrates the proposed mechanism of release from processed Dex-TEG-Dex implants. To experimentally demonstrate the mechanism by which Dex-TEG-Dex implants serve as drug delivery vehicles, Dex-TEG-Dex was heat-molded into pellets of 1 mm height and 1 mm diameter (Fig. 4a) and incubated in different media under sink conditions, including fetal bovine serum (FBS), 1% FBS in phosphate-buffered saline (PBS; pH 7.4), and PBS (pH 7.4) (Fig. 4b). In all media, pellets demonstrated controlled release in contrast to the typical burst release profiles associated with polymer encapsulation-based drug release systems^38,39^. Tracking of pellet morphology in FBS over time demonstrated a surface dissolution mechanism. The pellet size decreased over time as dimer was released from its surface, while the inner mass of the pellet remained in dimer form (e.g., no autocatalysis which is typical of PLGA) without degradation to free drug (Fig. 4c, Supplementary Fig. 5). The release rate of the dimer implants increased in higher protein content media. Release in 3.6% BSA in PBS (pH 7.4) was faster than in PBS alone. In contrast, the release rate of the dimer implants in 3.6% BSA was slower than in 100% FBS, despite having similar total protein content (Fig. 4d). Furthermore, while in 100% FBS minimal amounts of solubilized Dex-TEG-Dex dimer were detected, a higher proportion of the released products detected in 3.6% BSA were in the dimer form rather than in the free drug form. This difference in dimer prevalence in 3.6% BSA vs. 100% FBS, and the reverse trend for free drug, is hypothesized to be due to the presence of greater enzymatic activity in 100% FBS (e.g., butyrylcholinesterase, acetylcholinesterase^40,41^) which is reported to promote hydrolysis and would not be present in bovine serum albumin alone. This demonstrates that while passive hydrolysis plays a role in dimer degradation to free drug in BSA and PBS, this process is accelerated in the presence of enzymes (i.e., enzymatic activity within FBS). As expected for a surface dissolution phenomenon, drug delivery duration is well correlated to the diameter of formed devices. This is demonstrated by the drug release profiles associated with implants of different diameters (Fig. 4e). Collectively, these data support the hypothesis of surface dissolution for the dimer from the implant surface, followed by hydrolysis of the Dex-TEG-Dex dimer to give controlled release of free Dex.

### Degradation of solubilized Dex-TEG-Dex in vitro

Dex-TEG-Dex was designed to have minimal and well-defined degradation products (Dex-TEG, Dex, TEG, CO_2_), allowing for simple tracking of degradants as the materials are broken down. Incubation of solubilized Dex-TEG-Dex in FBS indicates that the dimer is converted to Dex-TEG and free Dex, with Dex-TEG subsequently hydrolyzed to yield free Dex (Dex-TEG-Dex *t*_1/2_ = 1.6 ± 0.3 h) (Fig. 4f). To assess the role of enzymatic activity in dimer degradation, studies were conducted in the presence of a protease inhibitor, which significantly extended Dex-TEG-Dex half-life (*t*_1/2_ = 17.5 ± 4.0 h) (Fig. 4g), highlighting the implication of enzymatic hydrolytic activity in the degradation of the solubilized dimers to free drug.

### Activity of dexamethasone released from degraded Dex-TEG-Dex

Activity of the Dex released from Dex-TEG-Dex was tested in vitro using a model of lipopolysaccharide (LPS) induced monocyte activation with human monocytes isolated from peripheral blood of healthy volunteers^42,43^ (Fig. 4h). A dose–response curve of free drug released from solubilized Dex-TEG-Dex was compared to free Dex. No differences were observed in Dex activity (Dex from Dex-TEG-Dex IC_50_ = 0.93 ± 0.49 nM, Dex = 1.52 ± 1.24 nM^44^), indicating that free drug released from Dex-TEG-Dex had equivalent activity to the parent drug.

### Design factors for controlling drug release from corticosteroid dimer materials

**Fig. 5:**
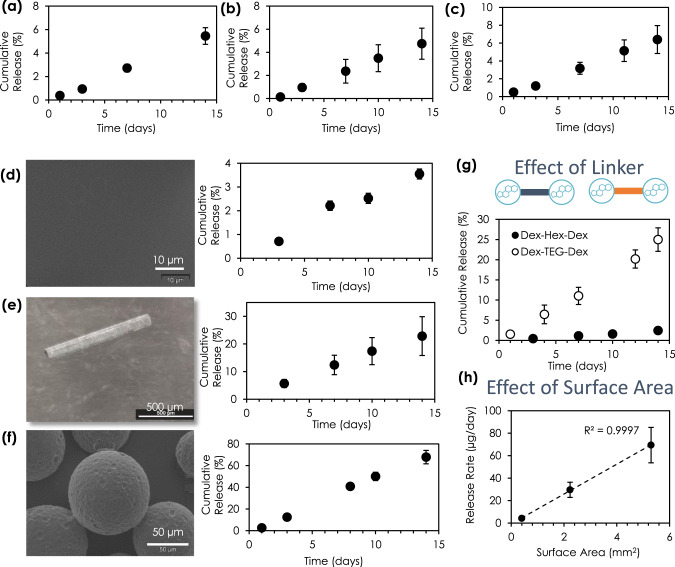
Versatility of the dimer material platform to achieve rational design. Release kinetics of heat-molded 1 mm×1 mm (height x diameter) pellets for free **a** prednisolone (Pred) from Pred-TEG-Pred (N=3 samples, TEG – triethylene glycol) in 4 ml phosphate buffered saline (PBS) (0.96 ± 0.1 mg Pred loading), **b** triamcinolone acetonide (TA) from TA-TEG-TA (*N* = 5 samples) in 4 ml FBS (0.81 ± 0.18 mg TA loading), and **c** hydrocortisone (HC) from HC-TEG-HC in 4 ml PBS (0.60 ± 0.08 mg HC loading) (N=3 samples). Scanning electron micrograph (SEM) images and release curves demonstrating free drug release for dimers processed into **d** coatings (4 ml PBS, 0.78 ± 0.03 mg dexamethasone (Dex) loading, 12 mm diameter circular coating) (*N* = 3 samples), **e** extruded rods (4.4 ml PBS, 17.7 ± 4.4 µg Dex loading) (*N* = 3 samples), and **f** microparticles (10 ml fetal bovine serum (FBS), 42.2 ± 18.2 µm diameter, 0.12 ± 0.01 mg TA loading) (*N* = 3 samples). For coatings, the SEM image indicates a smooth coating surface at high magnification. **g** Release rate in FBS for free Dex from Dex dimers with either a TEG or hexane diol (Hex) linker (2 ml, 1 mm×1 mm height x diameter pellets, 1.39 ± 0.27 mg Dex loading for Dex-Hex-Dex and 0.51 ± 0.05 mg for Dex-TEG-Dex) (N=3 samples). **h** Correlation between release rate and surface area for implants in FBS (1.5 ml FBS, 23 G × 6 mm−348 ± 141 µg loading, 30 G × 1 mm – 11 ± 2 µg loading, 30 G × 6 mm – 63 ± 16 µg loading). *N* = 3 samples. In all panels, data represent the mean ± standard deviation.

Next, we sought to understand the versatility of the dimer implant system by assessing the drug release function achieved with corticosteroids other than Dex, the use of different form factors, the effect of linker chemistry, and the capacity for delivery of two drugs simultaneously. Dimers were formed using different corticosteroids, demonstrating the ability to process dimers into physical forms that provide surface erosion-based drug release for drugs other than Dex, including release of prednisolone (Pred, Pred-TEG-Pred, Fig. 5a), triamcinolone acetonide (TA, TA-TEG-TA, Fig. 5b), and hydrocortisone (HC, HC-TEG-HC, Fig. 5c). Since different physical forms may be required depending on specific clinical indications (e.g microparticles for intraarticular administration, coatings to improve implantable device functional lifetime)^9,16^, corticosteroid dimers were manufactured into different forms. Sustained-release kinetics in each of these forms was confirmed, including coatings (Dex released from Dex-TEG-Dex, Fig. 5d), rods (Dex released from Dex-TEG-Dex, Fig. 5e), and microparticles (TA released from TA-TEG-TA, Fig. 5f, Supplementary Fig. 6). Release rate, and hence duration, was modulated using the same corticosteroid by the choice of linker. A more hydrophobic hexane diol linker significantly slowed the release of Dex relative to the more hydrophilic TEG linker (Fig. 5g). Based on the surface dissolution mechanism of drug release, it was hypothesized that dose could be controlled through an increase in surface area. Dex-TEG-Dex samples of different surface area demonstrated a predictable correlation between surface area and dose (*R*^2^ = 0.9997) (Fig. 5h). This allows implant dimensions to be tailored to target the desired dose and duration required for a specific indication or application.

**Fig. 6:**
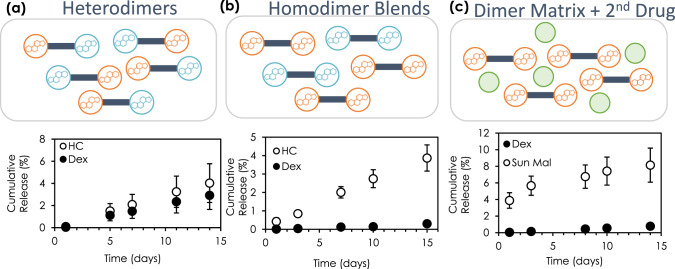
Formulation strategies for achieving multi-drug release with corticosteroid dimer materials. **a** Release kinetics for Dex and HC from a Dex-TEG-HC heterodimer (4 ml PBS, 1 mm × 1 mm height × diameter pellets, 0.18 ± 0.08 mg HC and 0.19 ± 0.08 mg Dex loading) (*N* = 3 samples). **b** Processing of two different corticosteroid homodimers (Dex-TEG-Dex and HC-TEG-HC) into a solid implant that gives controlled release of both drugs. (4 ml PBS, 1 mm × 1 mm height × diameter pellets, 0.51 ± 0.05 mg Dex and 0.35 ± 0.03 mg HC total loading) (*N* = 3 samples). **c** Use of a dimer (Dex-TEG-Dex) to provide a matrix for the support, distribution, and controlled release of Dex and a second drug (sunitinib malate). Dex-TEG-Dex:Sunitinib malate 7:1. (4 ml PBS, 1 mm × 1 mm height × diameter pellets, 0.83 ± 0.14 mg Dex and 0.15 ± 0.02 sunitinib malate loading). *N* = 3 samples. In all panels, data represent the mean ± standard deviation. Orange and blue circles represent different corticosteroids. Dark blue rectangles indicate the linker. Green circles represent free drug contained within the dimer matrix.

Next, we sought to evaluate the capacity of drug dimer materials to simultaneously deliver a second drug. First, a heterodimer of HC-TEG-Dex was synthesized. This dimer was heat-processed into a pellet and satisfied the criteria to yield formed devices with controlled release of both drugs (Fig. 6a). Physical blends of two homodimers (Dex-TEG-Dex and HC-TEG-HC) were also processed into a sustained-release implant, giving sustained release of HC and Dex (Fig. 6b). As stand-alone materials, Dex-TEG-Dex has a slower dissolution profile and thus drug release profile relative to HC-TEG-HC, which is also seen with the blended pellets of the two dimers, where release of Dex is slower relative to release of HC. This data suggests that when two dimers are combined (e.g., the 1:1 ratio employed in this study) that their inherent drug release characteristics will be maintained relative to one another (i.e., the slower releasing formulation will also release slower in the blended dimer pellet). The use of a dimer matrix to encapsulate and control the release of a non-covalently bound second drug also was demonstrated by preparing physical blends of sunitinib malate with Dex-TEG-Dex at a free drug:dimer ratio of 1:7. The dimer matrix provided sufficient stability to maintain form of the heat-processed pellet. An initial burst release of sunitinib malate is observed over the first three days of the release study (Fig. 6c). This initial burst is consistent with an initial solubilization of the water-soluble sunitinib malate on the exterior surface of the heat-molded pellet that is not fully encapsulated by the dimer, and is reflected in a decrease in the ratio of sunitinib malate to Dex-TEG-Dex from 1:7 at fabrication to 1:9 following recovery at the end of the study (Supplementary Fig. 7). Following the initial burst, however, the release rate of sunitinib malate is consistent between day 3 and day 14 and was controlled by the release rate of the dimer matrix. This allows for long-term drug release kinetics to follow kinetics associated with a surface erosion mechanism of drug release (Fig. 6c).

### Anti-inflammatory activity of a Dex dimer implant in a rat model of uveitis

**Fig. 7:**
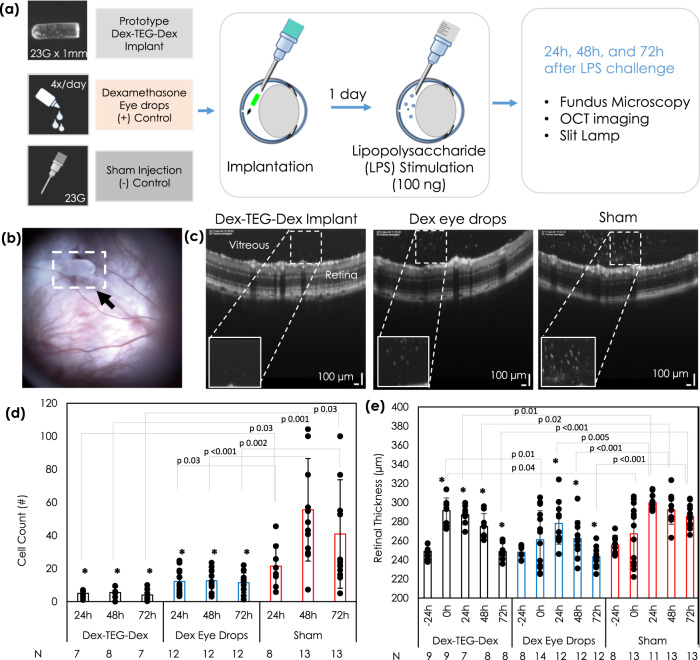
Acute efficacy of a dexamethasone dimer material in a rat model. **a** Experimental set-up demonstrating treatment groups, method of implant administration, and methods and timepoints for assessing efficacy. **b** Fundus micrographs of the Dex-TEG-Dex (Dex – dexamethasone, TEG – triethylene glycol) implant post-implantation in the vitreous humor. (**c**) Representative optical coherence tomography (OCT) images 24 h post-lipopolysaccharide (LPS) stimulation. **d** Cell counts in the vitreous humor following LPS stimulation for eyes with an intravitreal dexamethasone dimer implant, eyes receiving topical dexamethasone eyedrops, and eyes receiving a sham injection. Vitreal cell counts were performed on images obtained by OCT. **e** Quantification of retinal thickness from OCT images following LPS stimulation for eyes that received dexamethasone dimer implants, dexamethasone eye-drops, or a sham injection. Data represent the mean ± standard deviation. *N* numbers are indicated in the figure for parts d-e. Statistical analysis was performed by ANOVA. *p < 0.05 vs. sham injection.

To demonstrate the ability of a Dex-TEG-Dex implant to deliver active and efficacious levels of drug to treat a disease state, a lipopolysaccharide (LPS)-induced model of uveitis in a rat eye was employed (Fig. 7a). The inflammatory response induced by LPS has been shown to be inhibited by the presence of active Dex^45^. Prior to performing implant studies, a general cytotoxicity assessment was performed with human dermal fibroblasts to confirm that the Dex-TEG-Dex dimer would not be anticipated to cause a cytotoxic response (Supplementary Fig. 8). Dex-TEG-Dex implants were terminally sterilized by ethylene oxide. While residual ethylene oxide residue on implanted devices has a known potential for cytotoxicity, it remains a commonly used option for implanted devices, including commercially available intraocular implants. This is due to its compatibility with materials that may be sensitive to heat, moisture, or radiation exposure, provided that ethylene oxide residue levels (ethylene oxide and ethylene chlorohydrin) are maintained below values outlined in accepted standards for its use^46,47^. A Dex-TEG-Dex implant was injected into the intravitreal space of the rat eye using a 23 G needle (Fig. 7b), followed 24 h later by intravitreal injection of LPS to induce inflammation. Implants were shown to acutely suppress the LPS-induced inflammatory response, as demonstrated by the absence of cellular infiltrate and reduced retinal thickness (measured using optical coherence tomography (OCT)) relative to non-treated controls and with the same level of inhibition as topically administered Dex eye drops (Fig. 7c–e, Supplementary Fig. 9). These results indicate the ability of a processed Dex dimer implant to release efficacious levels of drug in vivo to counteract an inflammatory stimulus, with an effective dose sustained for the 72 h study period.

### Dissolution behavior of Dex dimer implants in the rabbit eye

The drug release characteristics for a Dex-TEG-Dex implant were next demonstrated in the rabbit eye. Diabetic macular edema, characterized by vascular leakage, tissue edema, and hard exudates on the retina, is a leading cause of visual impairment for diabetic patients^48^. Steroids are one method of treating this disease state, and since the target tissue (i.e., the retina) is located at the posterior of the eye, this is accomplished through the use of intravitreally administered corticosteroids and corticosteroid-releasing implants^16^. However, there is a need to provide sustained, low dose steroid delivery as high doses are associated with adverse side effects and currently available implants do not provide sufficient drug delivery duration^49^. Excessive drug levels lead to the presence of drug in off-target tissue sites (e.g., aqueous humor in the anterior chamber), which can result in adverse effects such as increases in intraocular pressure^18,50^. It was hypothesized that intravitreally administered Dex-TEG-Dex implants would undergo surface erosion, resulting in predictable, consistent release kinetics at the site of administration (vitreous humor) and in target tissue sites (retina), resulting in efficacious drug levels, while avoiding exposure in off-target tissue sites associated with adverse events (aqueous humor).

**Fig. 8:**
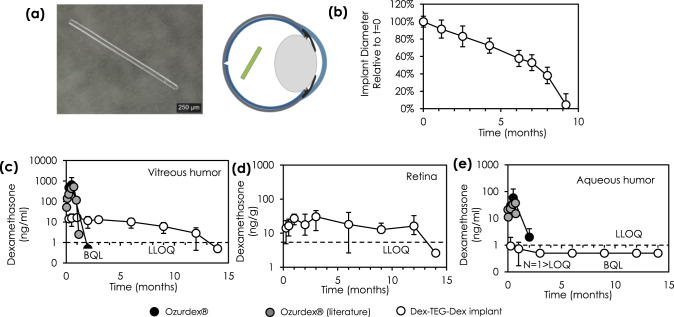
Dissolution and pharmacokinetics of a dexamethasone dimer implant following intravitreal administration in the rabbit eye. **a** Light micrograph of a 30 G x 6 mm heat-extruded dexamethasone dimer implant and schematic showing its physical location in the eye following intravitreal injection **b** Quantification of implant diameter over time from IR images for the 30 G × 6 mm Dex-TEG-Dex (Dex – dexamethasone, TEG – triethylene glycol) implants. *N* = 8 for all data points except *N* = 7 at 76 days. Data represent the mean ± standard deviation. Quantification of dexamethasone in the **c** vitreous humor, **d** retina, and **e** aqueous humor for 30 G × 6 mm dexamethasone dimer implants compared to experimental and literature^52,53^ findings for Ozurdex^®^ (vitreous humor only). *N* = 6 for the 30 G × 6 mm implant (except *N* = 5 for retina at 9 months) and *N* = 2 for Ozurdex^®^. Data represent the mean ± standard deviation. Dashed line indicates the lower limit of quantification for each tissue type.

To test this hypothesis, a 30 G × 6 mm extruded rod of a Dex dimer (Dex-TEG-Dex) was injected into the intravitreal space of rabbit eyes with a 30 G needle (Fig. 8a). Fundus and IR imaging (Fig. 8b, Supplementary Fig. 10) demonstrated the surface erosion phenomenon, with implant diameter decreasing over time. This surface erosion effect was also observed for a smaller diameter 32 G × 6 mm implant (Supplementary Figs. 10 and 11b). Using a mathematical model of drug release, experimental data was fitted to an equation derived to describe implant radius as a function of time to estimate a local surface dissolution rate constant. This constant, which describes the constant dimensional change that occurs as a result of the surface erosion-based mechanism of drug release, was determined to be 0.1510 ± 0.0084 µm/day for the 30 G implant and 0.1522 ± 0.0140 µm/day for the 32 G implant (Supplementary Fig. 11). This indicates that this rate constant is an intrinsic material property in the intravitreal space of the rabbit eye, independent of implant dimensions. This highlights the reproducibility and predictability of drug release from these materials, allowing for rational design of implant dimensions for target doses and durations. As a result, designing an implant with a desired duration is readily achieved due to the predictable scaling of duration with implant diameter (e.g., doubling implant diameter also doubles the duration of release). The dose delivered per day can also easily be predicted from the amount of drug contained in the volume that corresponds to this constant change in implant dimension over time.

### Pharmacokinetics of Dex dimer implants in the rabbit eye

Quantification of Dex following intravitreal administration of 30 G × 6 mm Dex-TEG-Dex implants was performed for the site of implant administration (vitreous humor), in the target posterior ocular tissue (retina), and in an off-target site in the anterior chamber (aqueous humor) (Fig. 8c–e). Slow, steady release of free Dex was observed in the desired target tissue sites (vitreous humor, retina), which fell below limits of detection at 14 months (Fig. 8c, d). Little to no drug was measured in the aqueous humor (Fig. 8e), an off-target tissue where the presence of corticosteroids could induce corticosteroid-associated adverse effects^50^. In contrast, Ozurdex®, a PLGA-based implant that is the market leader in corticosteroid-based intravitreal drug delivery (60:40 Dex:polymer ratio)^1,51^, experienced a burst release, with peak drug levels two orders of magnitude higher than the dimer implant at 1 month post injection and rapidly dissipated below detection limits shortly thereafter. Furthermore, elevated drug levels were found in the anterior chamber for Ozurdex®, an off-target site, while levels of dexamethasone in the aqueous humor for Dex-TEG-Dex were below the limit of quantification for most samples^52,53^. A well-documented side effect of intraocular use of corticosteroids, including dexamethasone, is an increase in intraocular pressure^54^. This is also reported clinically with Ozurdex®^18^. This increase in intraocular pressure is primarily caused by an increased resistance to the outflow of aqueous humor. While the precise mechanism causing increased resistance to outflow is unknown, the most widely regarded hypotheses implicate the presence of corticosteroids in the front of the eye causing cellular (e.g., endothelin-1 expression^55^) and tissue changes (e.g., decreased extracellular matrix turnover in the trabecular meshwork^56^) that increase outflow resistance. By significantly reducing the presence of dexamethasone in the aqueous humor relative to Ozurdex®, it is anticipated that Dex-TEG-Dex may minimize the potential for intraocular pressure spikes.

### Pharmacodynamics of Dex dimer implants in the rabbit eye

**Fig. 9:**
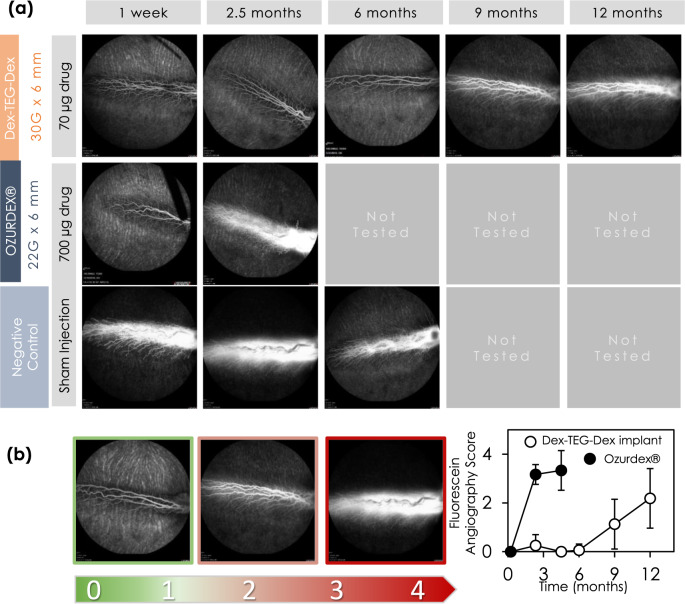
Pharmacodynamics of an intravitreal dexamethasone dimer implant in the rabbit eye. **a** Fluorescein angiograms of the back of the eye following intravitreal injection of vascular endothelial growth factor (VEGF) for eyes receiving 30 G × 6 mm dexamethasone dimer implants compared to Ozurdex® and a negative control (sham). **b** Semi-quantitative analysis of scoring for inhibition of VEGF-induced changes in the retinal vasculature. Data represent the mean ± standard deviation. *N* = 3 for Ozurdex® and *N* = 8 for 30 G × 6 mm Dex-TEG-Dex implants.

The efficacy of the Dex-TEG-Dex implants in the rabbit eye was assessed for their ability to counteract the effects of vascular endothelial growth factor (VEGF) induced blood-retinal barrier breakdown^57^ and compared to the performance of the Ozurdex® intravitreal implant. VEGF is a key mediator in the development of diabetic macular edema and, in this model, injection of VEGF induces blood vessel tortuosity, retinal blood vessel vasodilation, and increased vascular permeability, as visualized by fundus microscopy and fluorescein angiography. This effect can be inhibited by the administration of corticosteroids, including Dex^57^. In this model, the leading commercial implant, Ozurdex®, which is a PLGA-based implant containing free Dex, demonstrated efficacy at 1 week but was non-efficacious and indistinguishable from the negative control by 10 weeks (Fig. 9a, b), which reproduces the pre-clinical literature data for this product^52^. In contrast, 30 G × 6 mm Dex-TEG-Dex implants were efficacious at an acute time point of 1 week, which was maintained beyond 6 months before losing efficacy between 9 and 12 months (Fig. 9a, b), thereby extending effective doses well beyond times reported for traditional polymer-loaded Dex systems^51^. These data highlight the paradigm shift that is achieved with the drug dimer material technology. As a result of higher drug loading capacity and efficient drug delivery, an implant with one tenth the drug load of a polymer-based system is able to deliver efficacious levels of drug for at least three times longer (e.g., 9–12 months for 30 G × 6 mm Dex-TEG-Dex, vs. <2.5 months for Ozurdex®).

The correlation between implant diameter and duration of efficacy was demonstrated through further experiments with 32 G × 6 mm and 27 G × 6 mm Dex-TEG-Dex intravitreal implants. Similar to 30 G × 6 mm Dex-TEG-Dex implants, smaller diameter 32 G × 6 mm Dex dimer implants inhibited VEGF-induced effects at 1 week out to 4.5 months, but demonstrated reduced efficacy at 6 months (Supplementary Fig. 12). Larger diameter 27 G × 6 mm implants were efficacious at all time points tested, out to 18 months (Supplementary Fig. 12). These results confirm the expected correlation between duration and diameter for a surface erosion-based system. Key properties of drug dimer materials, namely surface erosion-based release kinetics and the use of physical parameters to rationally design the drug dose and duration, were thus shown to translate in vivo, and enable the predictable performance of an implant for a specific target dose and duration. Further, the technology yields the additional benefit of not generating acidic polymeric by-products, an inherent feature in the chemistry of PLGA and other degradable polyesters^51^.

### Safety assessment of intravitreal Dex-TEG-Dex implants

Intravitreal implantation has the potential to cause toxicity due to foreign body presence alone, as well as due to the presence and concentrations of drugs and degradation products in the case of biodegradable implants for drug delivery.

**Fig. 10:**
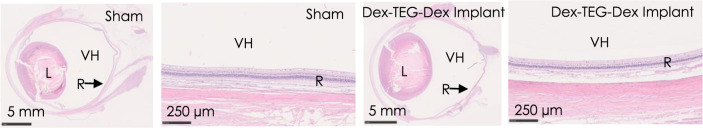
Ocular histopathology following intravitreal implantation of a dexamethasone dimer implant in the rabbit eye. Histology (hematoxylin and eosin (H&E)) of a rabbit eye 12-months post-intravitreal sham procedure or administration of Dex-TEG-Dex implants. VH – vitreous humor, L – lens, R – retina.

Assessments were performed to assess safety factors related to the physical presence of the implants and their released products, including the free drug dexamethasone and the TEG linker, following intravitreal implantation of 30 G × 6 mm Dex-TEG-Dex implants. In order to provide an additional safety factor with respect to both the presence of the implant and drug and linker release levels, the study compared groups receiving sham injections to eyes that received two or three 30 G × 6 mm Dex-TEG-Dex implants. Based on the surface erosion mechanism of release demonstrated for these materials (Figs. 3, 4c, 8b, Supplementary Figs. 11 and 12), two and three implants would be anticipated to reflect a dose-response with increased free drug and linker levels of two and three times, respectively, relative to a single implant of the same shape, size, and drug loading. Ocular toxicity was assessed through histopathology, which is considered the gold standard for assessing ocular toxicity^58^, as well as measurements of intraocular pressure and fundus microscopy to assess retinal changes. No effect of the presence of the Dex-TEG-Dex implants or their released products with respect to these parameters was observed over 52 weeks (Supplementary Fig. 13). IR fundus images, a measure of retinal health, of sham, two implant, and three implant eyes were unremarkable at baseline and 3, 6, 9, and 12 months post-implantation (Supplementary Fig. 13). Histopathology analysis (Fig. 10) indicated no microscopic or macroscopic findings that would suggest biocompatibility issues with the Dex-TEG-Dex implants, based on analysis performed by a board-certified veterinary pathologist. Thus, two or three Dex-TEG-Dex implants, simultaneously implanted intravitreally in rabbit eyes, were well tolerated out to the end of the experiment (12 months), demonstrating the safety of the Dex-TEG-Dex implants as foreign bodies in the intravitreal space as well as their released products as they undergo degradation and release free drug.

In contrast to Dex, the ocular toxicology of TEG has not previously been studied. However, the toxicological profile of TEG has been described in the literature and includes studies to evaluate local toxicology (topical skin application and skin contact, inhalation, eye contact, and skin sensitization), general toxicology (acute and chronic), developmental toxicology, reproductive toxicology, and genetic toxicology^59^. In these studies, no significant adverse toxicity has been identified by systemic routes of administration^59^. While ocular toxicity of TEG or the direct use of TEG in ocular products has not been identified, several low molecular weight PEGs are used in commercial ocular products that are given on a daily basis (e.g., PEG300, PEG400). These low molecular weight PEGs contain small amounts of TEG due to the polydispersity inherent to the PEGs at levels up to 0.2% for PEG300 and 1% w/v for PEG400, corresponding to maximum daily exposure levels of 0.43 and 2.16 mg PEG, respectively, based on recommended daily instillation requirements^60,61^. Comparatively, Dex-TEG-Dex implants release <0.00018 mg of TEG into the eye per day.

Due to the inherent characteristics of the individual components of Dex-TEG-Dex, such as high aqueous solubility (e.g., TEG), and the different dosing kinetics produced by administering bolus injections of components at high initial concentrations that would otherwise be released in a slow, consistent manner from the implants, the study of the individual components would introduce perturbations that complicate and confound the interpretation of the data. Therefore, the most relevant data to support the safety of the implants and their degradation components are safety assessments performed during the in vivo studies, which did not indicate any safety concerns at two or three times single implant dose levels (Fig. 10, Supplementary Fig. 13).

The advantages of sustained drug delivery are widely recognized and have been explored in the context of medicine since at least the 1960’s^5^. These advantages include increasing patient compliance; consistent targeting of therapeutic windows; and spatial targeting of the drug to desired sites of activity, thus minimizing systemic and other off-target side effects^5,19^. While the properties of the ideal drug delivery system have been apparent since the 1980’s^5^, the development of systems that can achieve all of these objectives remains a challenge.

From a safety perspective, an ideal drug delivery system would have well-defined and well-tolerated degradation products, be fully biodegradable, and not elicit an adverse tissue response^5^. Traditionally, the field has relied on the use of high molecular weight polymers, both biodegradable (e.g., polyesters such as PLGA) and non-degradable (e.g., silicone), to provide structural integrity to drug delivery systems encapsulating low molecular weight drugs^19,21^. In this study, the formation of implants to provide the controlled release of corticosteroids is demonstrated with the use of low molecular weight conjugates, without the requirement for binding agents or excipients to maintain structural integrity (Figs. 1b, 2d, e). As a result, the duration of drug release is synched completely with device degradation, without the possibility of implant material remaining longer than the drug release phase. This is a desirable characteristic in a drug delivery system since the physical presence of a foreign material perturbs the local environment, and has the potential to cause physical damage through contact with local tissues, as well as promoting inflammation and toxicity through the release of degradation products^62^. Minimizing the duration of foreign material presence to the period of drug release thus minimizes the potential for implant-associated adverse events. The long-term presence of physical implants can result in tissue fibrosis, and degradation products have been implicated in local pH changes, alterations in tissue cell phenotype, and alterations in gene expression^63–65^. Non-degradable polymer systems, such as ethylene-vinyl acetate^66^, require implant removal, with the associated risks inherent to any surgical procedure. In other cases, due to inaccessibility of implant location (e.g., in the posterior cavity of the eye), the device remains for the lifetime of the patient, which poses unnecessary risks for long-term, chronic adverse events. In the case of degradable polymer systems, polymeric materials that remain following completion of drug release can cause adverse events^67^.

Corticosteroid dimers can be synthesized as monodisperse entities, limiting the possible degradation products to a few components (free drug, linker, and drug-linker), as demonstrated through half-life studies with Dex-TEG-Dex (Fig. 4f). The ability to synthesize corticosteroid dimers as monodisperse entities enables reproducible manufacturing of the dimer and parameters for downstream manufacturing into their final processed forms. The use of standard melt extrusion processes for the fabrication of implants reduces the need for costly, specialized manufacturing processes. In contrast, polymers are typically synthesized as polydisperse entities, and can be degraded at multiple sites along the polymer backbone. This can generate degradation products with a diverse range of molecular weights and compositions that can be more difficult to characterize^21,68^. One of the most common degradable polymers employed for drug delivery is PLGA, which generates acidic degradation products that can decrease local pH, eliciting an inflammatory response and in some cases impacting the phenotype of cells in the local tissue^63,65^.

Additional features proposed for the ideal drug delivery system relate to performance, including providing consistent drug release kinetics; releasing drug through a surface-mediated process, such that bulk changes to the implant are not occurring during the degradation process; and the ability to simply and predictably modify the implant to yield desired doses and durations^5^. Following exposure to aqueous environments and biological media, corticosteroid dimer implants undergo surface erosion, providing controlled release of free drug in both in vitro release studies (Figs. 3, 4b–e, 5a–h, 6a–c) as well as in the form of intravitreal implants in rabbits (Fig. 8b–e). Through the drug release process, the bulk of the implant remains in dimer form and does not degrade to free drug, providing further evidence of the drug release process being surface-mediated (Supplementary Fig. 5). This is in contrast to the often observed bulk erosion mechanism of release associated with conventional degradable polymer systems, such as PLGA^68,69^. In these systems, water penetrates the polymer matrix, resulting in swelling and subsequent degradation of the hydrolyzable bonds in the polyester backbone. The release of acidic degradation products within the polymer matrix can further catalyze the degradation process (i.e., autocatalysis), resulting in heterogeneity in polymer degradation due to the development of pH gradients^65^. The release of entrapped drug within these matrices is thus dependent on diffusion of drug out of the polymer, which changes over time as the material degrades and polymer chains are cleaved, as well as erosion occurring at the surface of the material^21,70^. This typically results in inconsistent, burst, and multi-phase release kinetics that are inherently variable and unpredictable in their already complex in vivo environments^21,71^.

Modifying drug release kinetics for degradable polymer systems can be achieved through changes to the chemistry of the polymer backbone, polymer molecular weight, and the choice of degradable linkages with differing hydrolytic or enzymatic susceptibility^5^. However, this approach can often yield non-intuitive changes to release kinetics^21,72^, requiring significant experimental investigation first in vitro and subsequently in vivo to understand the impact of changes that have been made on release kinetics. In contrast, the systems presented in this work present a practical and simple approach to predicting performance, based on the surface-erosion mediated release kinetics of the corticosteroid dimers. The result is that very simple changes in implant specifications can be used to change both dose and duration. With a surface erosion-based process, the dose delivered is proportional to the implant surface, and increases in surface area are shown to correspond to a predictable increase in dose delivery (Fig. 5h). Furthermore, drug delivery duration is directly correlated to the thickness of the implant (e.g., diameter for a cylindrical implant), a finding that was confirmed through in vitro release testing, as well as with intravitreal implants of a Dex-TEG-Dex implant of different diameters (Fig. 4e, Supplementary Fig. 12). Furthermore, linker chemistry can be used as an additional lever to modify the duration of release, with more hydrophobic linkers resulting in slower release kinetics (Fig. 5g).

In summary, we have demonstrated a transformative material concept in the field of drug delivery. The chemical changes implemented in the dimer molecules effectively provide their own vehicle for controlled drug delivery, dispensing with a decades-old paradigm of needing a second phase material to achieve long-term controlled release of a drug. These drug dimer materials are formed solely from low molecular weight conjugates (MW~1000 Da) that can be readily processed into solid amorphous implants. We believe these findings have significant implications for drug delivery with the potential to shift how we think about designing materials for controlled drug release, and thereby enabling the field’s ability to treat numerous disease states with consistent and directed drug release for a specified duration.

## Methods

### Materials

Solvents were purchased from Caledon unless otherwise noted. Other in vitro reagents were purchased from Sigma-Aldrich unless otherwise noted. Drugs were purchased from various vendors, including Medisca (dexamethasone, hydrocortisone, prednisolone, triamcinolone acetonide) and LC Labs (sunitinib malate). Cells and reagents for cell culture were purchased from Cell Applications.

### Synthesis

Glucocorticoid steroids were selectively reacted through the C-21 hydroxyl to give steroid-linker-steroid dimers with either carbonate or ester hydrolyzable bonds. Carbonates were synthesized via diol bis(chloroformate)s and esters via carbodiimide coupling reactions with dicarboxylic acids. In a typical procedure the steroid (1 mol. equiv.) was suspended in dichloromethane at 0 °C and triethylamine (2 mol equiv.) and triethylene glycol bis(chloroformate) (0.6 mol equivalent) were added to the mixture. The ice bath was allowed to warm to room temperature and the reaction was stirred overnight. The solvent was removed and the solid residue was purified by column chromatography. Product was recrystallized from organic solvent.

Non-symmetrical dimers were synthesized as follows: Drug 1 was treated with 4-nitrophenylchloroformate to yield the activated carbonate which was reacted with triethylene glycol to generate the mono steroid-TEG carbonate. The latter product was then treated with the chloroformate of drug 2 to yield the drug1-TEG-drug2 non-symmetrical carbonate dimer. This product was then purified as described above.

### NMR

NMR of dimers was conducted with a Varian Mercury 400 NMR spectrometer (University of Toronto). NMR data for the dimers can be found in Supplementary Figs. 1 and 14–18.

### FTIR

Samples were prepared neat and spectra were obtained using a PerkinElmer Spectrum One (spectral range 7800–400 cm^−1^). For Dex-TEG-Dex, FT-IR (wavenumber cm^−1^) 3431, 2943, 2877, 1754, 1726, 1661, 1609, 1453, 1412, 1394, 1375, 1353, 1275, 1242.

### Mass spectrometry

High-resolution mass spectrometry (HRMS) was performed with an Agilent 6538 QTOF with an ESI MS+ ion source with high resolution, accurate mass capability. HRMS data are reported in the figure captions for Supplementary Figs. 1 and 14–18.

#### Preparation of dimer materials

*Pellets for release studies*. Crystalline drug dimers were weighed on a stainless steel coupon. In the case of blends with a second drug, the dimer and drug were mixed at the desired mass ratio with mortar and pestle prior to weighing. Samples were transferred to a heat press (Carver Laboratory Press). A PTFE mold containing an array of 1 mm diameter holes of 1 mm depth was placed on top of the melt, and pressure was applied for 2 min. Molded pellets were cooled to room temperature and punched out of the mold, yielding a pellet of 1 mm height × 1 mm diameter.

*Spray coating and electrospinning*. Dimers were dissolved in acetone or tetrahydrofuran at concentrations ranging from 100–1000 mg/ml depending on the study. The solution was injected at a rate of 0.5 ml/h onto a stationary aluminum collector plate. An 18 kV potential difference between the needle and collector plate was maintained, with the needle charge set at +17.5 kV and the collector at −0.5 kV.

*Solvent-based fiber pulling*. Drug dimers were dissolved in DCM at 1 g/ml. Forceps were used to pull the resulting mixture and generate fibers. Fibers were left to dry overnight at room temperature.

*Emulsion and microparticle fabrication*. Drug dimers were dissolved in dichloromethane at 25% w/v, and 200 µl of this solution was then added to 400 ml of an aqueous solution of 1% sodium dodecyl sulfate (SDS, Sigma-Aldrich) through a Shirasu Porous Glass membrane (SPG Technology Co., Ltd). Samples were stirred for 1 h at room temperature. Serial centrifugation was used to remove residual SDS and collect microparticles. Microparticles were re-suspended in 0.5% carboxymethylcellulose (CMC, Sigma) in Milli-Q water at a concentration of 1 g/ml. 50 μl of the microparticle suspension was deposited onto carbon tape and left to dry overnight. Once dry, the samples were sputter-coated with gold at a thickness of 15 nm. Images were obtained at ×300 magnification. Microparticle diameter measurements were obtained using ImageJ (v. 1.8.0), in which the diameters of the microparticles were determined by normalizing the scale bar on the images with the corresponding distance in pixels. All microspheres that were un-obstructed and completely visible in the images were measured. Four batches of microparticles were assessed, with a minimum 200 microparticles measured per batch.

To prepare nanoparticles, dimers were dissolved in DCM at 25% w/v, and 200 µl of the solution was added to 3.8 ml of 1% SDS and sonicated for 1 min. Samples were then washed and nanoparticles collected by serial centrifugation. Nanoparticle size distributions were assessed using a DelsaMax Pro (Beckman Coulter).

*Extruded rod fabrication*. Dimers were weighed on aluminum foil and transferred into a length of PTFE tubing. The material was then loaded into a brass (McMaster-Carr) piston extruder barrel pre-heated with a collar heater & controller (Omega CSi32k). The extruder motor, a syringe pump (New Era) advanced a PTFE ended 1 mL Hamilton syringe piston, driving the molten dimer from the brass die (3D printing nozzle, E3D), onto a custom linear, tensioned belt, manually controlled variable-speed draw-down system to provide further control of the rod diameter. Extrusion and draw-down rates were adjusted as needed to compensate for selected extrusion die diameter, and drug-dimer material properties.

*Solvent casting*. Dimers were dissolved in acetone at 100 mg/ml. 10 µl of the solution was cast onto a 1 cm × 1 cm stainless steel or glass coverslip and left to dry at room temperature overnight.

*Powder coating*. Electrosprayed samples were heated at 185 °C to yield a smooth conformal coating.

*Heat-based fiber pulling*. Crystalline drug dimers were weighed on a stainless steel coupon and transferred to a heated stainless steel block. After fully melted, forceps were used to pull the melt solution, yielding fibers.

### SEM

Samples were placed on carbon tape and coated with 10 nm gold using a Leica EM ACE sputter coater. Coated samples were imaged using a FEI XL30 SEM with an accelerating voltage of 10 kV.

### TEM

A 5 µl droplet of nanoparticles at ~0.2 mg/ml was placed on a continuous carbon-coated copper grid (200 mesh). Excess water was removed by blotting with filter paper from the bottom of the grid, and left to dry overnight at room temperature. Samples were imaged with a Philips CM10 TEM.

### Stability study

Extruded Dex-TEG-Dex implants were assessed for shelf-life stability. Melt extruded Dex-TEG-Dex materials were packaged in the lumen of 30 G needles (BD) and stored in an anhydrous pouch containing desiccant. Samples were aged at 25 °C, 40 °C, and 70 °C for up to 4 months, corresponding to real-time aging of 126 days (25 °C, 4 months), 356 days (40 °C, 4 months), and 2851 days (70 °C, 4 months). At specified time points, samples were removed from their packaging and assessed by polarized light microscopy as outlined below.

### Polarized light microscopy

Samples were placed on glass slides, cross-sectioned as needed and imaged with a polarizing light microscope (SM-LUX-POL, Leitz) equipped with a Leica EC4 digital camera (Leica) using LAS EZ v.3.2.0 software (Leica). Additionally, a λ-plate compensator was used to assist in evaluation of features with weak birefringence.

### Differential scanning calorimetry

5–10 mg of each sample was heated from −20 °C to 220 °C at 10 °C/min, with experiments carried out using a DSC Q100 (Thermal Analysis).

### PXRD

Samples were analyzed with a Bruker D8 Discover (Bruker) with a Cu Kα source (Eurofins Alphora, Mississauga, Ontario, Canada). Results were processed using Diffrac.Eva software (version 4.2.1, Bruker). The diffraction patterns were integrated from two-dimensional to one-dimensional, and the background was subtracted.

### Mechanical testing

Extruded rods of ~300 µm diameter and 8 mm length (6 mm span on testing apparatus) were subjected to a 3-point bend test using an Instron uniaxial servo-hydraulic testing machine (Instron Model 8501) with a 2.5 N load cell and cross-head speed of 0.8 mm/min.

### HPLC

Calibration curves for the analysis of drug and precursor products were obtained on an Agilent 1260 Series HPLC Instrument (Agilent Technologies) configured with a quaternary pump (G7111B), vial sampler (G7129A) and diode array detector (G7117C). Agilent OpenLAB ChemStation Rev. C.01.07 software was utilized for system control, data acquisition and processing. Chromatographic separations were performed using a Phenomenex Gemini-NX C18 column (5 µm; 110 Å; 250 × 4.6 mm) equipped with a Security Guard Analytical Guard cartridge (Gemini C18; 4 × 3.0 mm). Temperature of the column was maintained at 25 °C, while the vial sampler was controlled to 6 °C. For all species, gradient elution with constant flow (1 mL min^−1^) was employed with mobile phases consisting of: (A) 0.05% trifluoroacetic acid (TFA) in H_2_O, and (B) 0.05% TFA in acetonitrile (MeCN). More specifically, gradient conditions followed the profile: (0 min 80% A), (40 min 16% A), (42 min 0% A), (50 min 0% A), (50.1 min 80% A), (60 min 80% A). Injections employed volumes of 5 µL for all tested constituents as well as calibration standards.

Stock solutions (2 mg mL^−1^) of Dex-TEG-Dex, Dex-TEG, Dex, Dex-TEG-HC, HC-TEG-HC, Hydrocortisone, Pred-TEG-Pred and Prednisolone were prepared in MeCN. Appropriate dilutions were made in MeCN to prepare standards (0.05–2000 µg mL^−1^) for all calibration curves.

Stock solutions (2 mg mL^−1^) of Dex-Hex-Dex and Dex-Hex were prepared in acetone. Appropriate dilutions were made in acetone (HPLC grade) to prepare standards (0.05–2000 µg mL^−1^) for the calibration curves.

Stock solutions (2 mg mL^−1^) of TA-TEG-TA, TA-TEG and Triamcinolone Acetonide were prepared in methanol (MeOH). Appropriate dilutions were made in MeOH to prepare standards (0.05–2000 µg mL^−1^) for the calibration curves.

Stock solution (2 mg mL^−1^) of sunitinib malate (SM) was prepared in dimethyl sulfoxide (DMSO, HPLC grade). Appropriate dilutions were prepared in DMSO to prepare standards (0.05–2000 µg mL^−1^) for the calibration curve.

### In vitro release

Release studies were carried out using different media, including PBS (pH 7.4, Gibco), 100% FBS (Sigma, F1051), 1% FBS in PBS pH 7.4, and 3.6% BSA (Sigma, A7906) in PBS (pH 7.4, Gibco). Release buffer volumes, total drug loadings, and sample dimensions are indicated in the figure captions. Sink conditions were maintained across all of the in vitro release studies through frequent buffer changes, ensuring that the released drug concentration was always at least five times lower than the solubility limit of the drug in the release buffer being used in order to avoid changes in release kinetics caused by drug levels approaching their saturation limit. Samples were incubated in release medium at 37 °C under constant agitation on an orbital shaker at 115 rpm (VWR). At designated timepoints, sampling was performed by complete removal of medium and replenishment with fresh buffer. Sampling timepoints included 24 h and then every 3–4 days thereafter. For PBS release conditions, samples were analyzed directly. For samples with protein (100% FBS, 1% FBS, 3.6% BSA), protein was precipitated by addition of acetonitrile. Samples were vortexed and centrifuged to separate extracted drug from precipitated protein. The supernatant was then analyzed for drug concentration by HPLC.

### In vitro half-life

Dex-TEG-Dex was dissolved in acetone at 10 mg/ml and added to 100% FBS to obtain a final concentration of 10 µg/ml and incubated at 37 °C with constant agitation at 115 rpm (VWR). At experimental timepoints of *t* = 0, 0.5, 1, 2, 3, 4, 5, 6, and 24 h, sample was removed and protein was precipitated with acetonitrile. Samples were vortexed and centrifuged to separate extracted drug from the precipitated protein. The supernatant was then analyzed by HPLC as described above.

### Cytotoxicity assessment

Human dermal fibroblasts (Cell Applications, 106-05A) were seeded at 5000 cells/well in a 96 well plate (BD). Human dermal fibroblasts were chosen for general cytotoxicity assessment as they are commonly employed in standard cytotoxicity testing outlined in ISO10993 guidelines^73^. Cells were incubated overnight at 37 °C and 5% CO_2_ prior to exposure to Dex-TEG-Dex at its maximum soluble concentration in culture medium (fibroblast growth medium, Cell Applications, cat #116-500) and 10-fold dilutions of this maximum concentration. This maximum concentration was achieved by dissolving Dex-TEG-Dex at 1 mg/ml in ethanol, and adding 100 µl of this solution to 9.9 ml culture medium (100x dilution) to achieve a theoretical concentration of 10 µg/ml. The resulting solution was sterile filtered (Pall) to remove any non-soluble components and the concentration of Dex-TEG-Dex was confirmed by HPLC (6.7 µg/ml). The positive control was regular culture medium with 1% ethanol, while the negative control was culture medium containing 5% DMSO. Following 24 h exposure to the different test groups, a WST-1 assay was performed. Briefly, WST-1 reagent (Roche) was mixed with growth medium at a 1:10 dilution, added to test groups, and incubated at 37 °C and 5% CO_2_ for 1 h. Supernatant was transferred to a new well and absorbance was measured at 450 nm. After 24 h exposure to test groups, samples were also visualized following Diff-Quik staining following the manufacturer’s recommended protocol (Siemens). Samples were imaged with a Leica DMIL microscope equipped with a Canon PowerShot S70 camera with ZoomBrowser Ex 5.0 software (Canon, version 5.0.0.142).

### Monocyte isolation, culture, and LPS stimulation

Monocytes were isolated from whole peripheral blood donated by healthy volunteers^19^. Protocols were approved by Health Sciences Research Ethics Board (REB) at the University of Toronto (ethics approval #22203). All experiments were repeated with three distinct donors who provided informed consent. Briefly, whole blood was layered on Histopaque-1077 (Sigma) and the mononuclear cell fraction was isolated by density centrifugation. Platelets were removed through a series of washes. Monocytes were seeded at a density of 200,000/well in a 96-well plate (Becton Dickinson). Medium was changed 2 h post-seeding to remove non-adherent cells. Following an overnight incubation at 37 °C and 5% CO_2_, medium was replenished and to each well 0.2 µl of stock Dex concentrations in DMSO were added to yield final concentrations in culture medium of 0, 0.1, 1, 10, 100, and 1000 nM. Dex derived from degraded Dex-TEG-Dex was prepared by dissolving Dex-TEG-Dex in ethanol at 10 mg/ml, adding to culture medium to achieve a concentration of 10 µg/ml, and subsequent incubation of the solution at 37 °C and 5% CO_2_ until the dimer had fully degraded, as confirmed by HPLC. Samples were diluted in culture medium to obtain concentrations of 0.1, 1, 10, 100, and 1000 nM Dex and added to wells containing monocytes. Samples were incubated for 15 min at 37 °C and 5% CO_2_, followed by addition of a 2 µl lipopolysaccharide stock solution (LPS, Sigma) to obtain a final concentration of 50 µg/ml. Following overnight exposure to LPS, supernatant was collected and analyzed for PGE_2_ using a PGE_2_ ELISA kit according to the manufacturer’s instructions (R&D Systems).

### Bioanalytical quantification

Drug quantification for rabbit ocular PK studies was performed at Intertek Pharmaceutical Services (San Diego, CA, USA) by LC-MS/MS. Analysis of samples was conducted using a SCIEX API 5000 mass spectrometer (Applied Biosystems/MDS Sciex), Shimadzu LC-20ADXR HPLC pumps, Shimadzu SCL-10A controller, and HTS-PAL autoinjector (CTC Analytics). Chromatography was performed using a Waters X-Bridge C-18 (3.5 µm; 100 × 2.1 mm) column equipped with a Phenomenex KrudKatcher ULTRA HPLC In-Line Filter (Part AF0-8497). The column was held at a temperature of 40 °C, while the autosampler compartment was maintained at 4 °C. Mobile phases consisted of: (A) 95:5 H_2_O:MeOH with 2 mM Ammonium Acetate (NH_4_OAc) and (B) 85:10:5 MeOH:H_2_O:MeCN with 2 mM NH_4_OAc. A gradient elution profile was employed, following the trend of: (0 min 30% B), (0.50 min 30% B), (2.50 min 65% B), (2.51 min 100% B), (4.50 min 100% B), (4.51 min 30% B), (6.50 min 30% B). Flow was constant at 0.6 mL min^-1^ with an injection volume of 10 µL. For MS parameters, the system was operated in positive ion mode with a TurboIonSpray probe monitoring transitions (±0.2 AMU) of m/z 393.2 → 373.2 for Dex and 397.3 → 377.2 for the Dex-d_4_ deuterated internal standard. Analyst Version 1.4.2 (Applied Biosystems-MDS Sciex) was used for instrument control, data acquisition, and peak integrations, while calculations including peak area ratios, standard curve regressions, sample concentration values, and descriptive statistics were performed with the assistance of the ALIS2004 validated LIMS.

Dex and internal standard (Dex-d_4_) were extracted from vitreous humor on ice using protein precipitation. Samples were initially centrifuged at approximately 1500 rcf for 5 min to remove any interfering particulates. Stepwise, the protocol for extraction from vitreous humor was to (1) pipette 50 µL of vitreous humor into an extraction tube; (2) add 13 µL H_2_O and vortex to mix; (3) add 25 µL internal standard spiking solution (100 ng/mL Dex-d_4_ in a 1:1 solvent of MeOH:H_2_O); (4) add 25 µL of make-up reagent (1:1 mixture of MeOH:H_2_O); (5) vortex mix for 5-10 s at a speed setting of 4 or 5; (6) centrifuge at ~60 rcf for 2 min; (7) add 300 µL of 9:1 MeOH:5 mM NH_4_OAc at pH 7 to precipitate proteins; (8) cap and vortex mix at full speed for 2 min; (9) Centrifuge at approximately 2700 rcf for 5 min; (10) transfer 300 µL of supernatant to a separate tube; (11) vortex the collected supernatant for 30 s at a low to moderate speed; (12) centrifuge at ~1500 rcf for 5 min; (13) store prepared extract at 4 °C until analysis by LC-MS/MS.

#### Rat uveitis model

*Sample preparation*. Dex-TEG-Dex was weighed on a stainless steel coupon and transferred to a heat press (Carver Laboratory Press). Once melted, a polytetrafluoroethylene (PTFE) template was placed on the melt and pressure was applied for 2 min. Samples were cooled to room temperature prior to removal from the PTFE mold, yielding implants with 23 G diameter × 1 mm length. Samples were loaded into appropriate-sized needles (23 G), packaged, and sterilized by ethylene oxide gas.

*LPS stimulation and implantation*. Animals (Brown Norway rats, female, 3–5 weeks old, 80–120 g) were handled in accordance with the animal research ethics board (AREB) at McMaster University as well as McMaster University guidelines for animal work (AUP# 15-03-09). Animals were first sedated in an isoflurane gas chamber before being anaesthetized through an intraperitoneal injection of ketamine (100 mg/kg) and xylazine (10 mg/kg) solution. One day prior to LPS stimulation, eyes were prepared with topical anesthetic eye drops (Alcaine, 0.5%, Alcon) and pupils were dilated with application of one drop of 1% tropicamide (Alcon) on each cornea prior to anesthesia. Implants were then injected with a 23 G needle into the vitreous humor of rat eyes. Negative control animals received a sham injection, while the positive control group received a sham injection as well as Dex eye drops 4× daily (Maxidex 0.1%, Alcon). One day after test article administration, each rat received an intravitreal injection of 100 ng of LPS (LPS *Escherichia coli* 0111:B4, Sigma Aldrich) to induce inflammation. The inflammatory state of the eyes was monitored by slit-lamp microscopy (Phoenix Anterior Segment Slit Lamp), fundus microscopy (Phoenix Micron IV), and optical coherence tomography (Phoenix image-guided OCT) for 72 h post-LPS injection. Slit-lamp was used to image the anterior structures of the rat eye and to assess changes in vasculature, aqueous humor haze, pupil responsiveness, and presence of cellular infiltrates. Anterior segment OCT (ASOCT) was used to assess signs of inflammation in the anterior chamber through anterior chamber flare, pupillary fibrin occlusions, and presence of inflammatory cells. The ASOCT was used only for qualitative evaluation of the inflammatory state. Posterior segment OCT (PSOCT) was used to capture six (6) images around the optic disc (OD) of each eye and the images were used for retinal thickness, inflammatory cell count, and vitreal haze evaluation. Retinal thickness was measured using ImageJ (v.1.8.0) from four (4) OCT images close to the optic disk. Inflammatory cells were manually quantified from the same OCT images used for retinal thickness measurements.

#### Rabbit model

*Sample preparation*. All implants were formed by melt extrusion of Dex-TEG-Dex, followed by cutting of implants to desired length. Samples were loaded into appropriate sized needles (27 G for 27 G implant, 30 G for 30 G, and 32 G implants), packaged, and sterilized by ethylene oxide gas.

*IR imaging*. Wide-angle infrared (IR) imaging was performed at baseline, and monthly thereafter for implant imaging and eye health assessment. The imaging was performed using a Heidelberg Spectralis® imaging platform and IR images were analyzed using image analysis software (Image J v.1.8.0) to measure the diameter change of the implants. Three diameter measurements per image and a minimum of two images per implant were used to determine the diameter of the implant at each time point.

*Pharmacokinetics*. Rabbit pharmacokinetic studies were performed at McMaster University (Hamilton, Ontario, Canada). Animals were handled in accordance with the animal research ethics board (AREB) as well as McMaster University guidelines for animal work (McMaster University AUP# 15-03-09). Animals were anesthetized with ketamine (up to ~50 mg/kg) and xylazine (up to ~10 mg/kg). Eyes were surgically prepared before intravitreal procedures with 5% betadine and topical anesthetic eye drops (proparacaine hydrochloride, 0.5%). Prior to test article administration, pupils were dilated with topical application of one drop each of 10% phenylephrine and 1% tropicamide on each cornea.

Implants (30 G × 6 mm Dex-TEG-Dex extruded rods, Ozurdex®) were injected (30 G needle for Dex-TEG-Dex, 22 G needle for Ozurdex®) intravitreally into the eyes of New Zealand White rabbits (female, 1.5–2.5 kg) with terminal timepoints of 1 and 2 weeks and 1, 2, 3, 6, 9, 12, and 14 months. Ocular tissues and plasma were collected at each timepoint and analyzed for Dex by LC/MS/MS. Whole globe eyes were collected into centrifuge tubes and remained frozen for a minimum of 24 h before dissection and tissue collection. During dissection, vitreous humor containing any remaining test article was collected frozen into pre-weighed centrifuge tubes and stored at −80 °C until further processing. All vitreous humor samples underwent a two-step centrifugation process to remove any remaining implant before tissue extraction and bioanalysis.

*Mathematical modeling of drug release*. Modeling of the Dex-TEG-Dex implants in the rabbit eye was performed using a compartmental model of drug release using MathWorks MATLAB R2020b. In this model, the drug delivery device is formed of solid drug dimer which undergoes surface erosion to release dimer into an intermediate domain. In the intermediate domain, dimer undergoes degradation to free drug and mono (linker-drug), which subsequently degrades to free linker and free drug. These components undergo transport to the target domain and are then cleared.

This model employs several simplifying assumptions. (1) The mechanism of surface dissolution is local (the same for each part of the surface independent of its location on the surface of the implant), in a manner dependent on the properties of the implant’s physiological location (e.g., dimer saturation). (2) Rate constants of processes are time independent, and the dissolution rates only depend on the corresponding changes in the shape of the delivery devices and properties of their environment. (3) Dimer degradation in the domain of interest occurs on a comparatively fast time scale such that the saturation concentration of dimer is not reached.

A differential equation for the radius, *R(t)*, of a cylindrical drug delivery device described by the compartmental model and derived using Noyes–Whitney equation^74,75^ can be written as (1):

1 $$\frac{{dR}}{{dt}}=-\frac{D{\mu }_{Y}}{\rho h}\cdot {y}^{\ast }=-b,$$

where *D* is a diffusion coefficient, *µ*_*Y*_ is the molecular mass of the dimer, *ρ* is the density of the drug delivery device material, *h* is the thickness of the concentration gradient layer near the surface, y* is the saturation concentration of the dimer and *b* is the local surface dissolution rate.

The initial radius, *R*, of the cylindrical device is known: $R\left(0\right)={R}_{0}$. Integrating Eq. (1) with this initial condition, gives Eq. (2):

2 $$R\left(t\right)={R}_{0}-b\cdot t$$

Equation (2) was fit to available data (time-dependent measurements of implant diameter) to estimate the local surface dissolution rate constant *b* for the 30 G × 6 mm and 32 G × 6 mm Dex-TEG-Dex intravitreal implants.

*Pharmacodynamics*. Rabbit pharmacodynamics studies were performed at Absorption Systems California (San Diego, CA, USA). All studies were conducted in compliance with relevant ethical regulations, including the USDA Animal Welfare Act (i.e., relevant sections of Section 9, Part 1, 2, and 3 of the Code of Federal Regulations) and in compliance with Absorption Systems’ Animal Welfare Assurance filed with the National Institutes of Health. Protocols were reviewed and approved by Absorption Systems’ Institutional Animal Care and Use Committee (IACUC) prior to the initiation of procedures. Treatment of animals was in accordance with conditions specified in the *Guide for Care and Use of Laboratory Animals* (NRC, 2011, National Academy Press). Animals were anesthetized with intramuscular ketamine hydrochloride (15-20 mg/kg) and xylazine (5 mg/kg) prior to test article injection or vascular leakage induction. The eye area was surgically prepped with 5% betadine and rinsing with balanced salt solution, followed by administration of 1–2 drops of topical proparacaine hydrochloride (0.5%). Prior to injection, pupils were dilated with one drop each of 10% phenylephrine and 1% tropicamide on each cornea. Test articles (27 G × 6 mm, 30 G × 6 mm, and 32 G × 6 mm Dex-TEG-Dex extruded rods, or Ozurdex®) were injected intravitreally in Dutch Belted rabbits (male, 1.84–2.49 kg, 5–6 months) on Day 0 and vascular leakage was induced by an intravitreal injection of VEGF (500 ng/eye) at acute and chronic timepoints (1 week, 2.5, 4.5, 6, 9, 12, and/or 18 months) post-implantation. Post-injection analgesia was provided by 0.02-0.05 mg/kg IM/SC buprenorphine. Negative control rabbits received a sham injection while positive control rabbits received a 2 mg solution dose of triamcinolone acetonide (Kenalog-40). Ophthalmic exams, including fundus microscopy and slit lamp microscopy, were carried out over the 3 days post-VEGF induction and vascular leakage was measured by fluorescein angiography on the third day post-VEGF induction. Eyes were graded based on fundus and fluorescein angiography images using a scoring system as follows^76^: 0 – major vessels straight, some tortuosity of smaller vessels, no vessel dilation; 1 – increased tortuosity of major vessels and/or some vessel dilation; 2 – leakage between major vessels, significant vessel dilation; 3 – leakage between major and minor vessels, minor vessels still visible; and 4 – leakage between major and minor vessels, minor vessels poorly/not visible.

*Tolerability study*. The tolerability study was conducted at Absorption Systems California (San Diego, CA, USA) under the same animal care and ethics guidelines and approval systems as indicated above in the *Pharmacodynamics* section. Female New Zealand White rabbits (2.73–3.83 kg) were assigned one of three treatment groups. Animals were anesthetized with intramuscular ketamine hydrochloride (30–32 mg/kg) and xylazine (5–10 mg/kg), with glycopyrrolate (0.01–0.02 mg/kg, intramuscular) administered concurrently. Eyes were prepped with 1–2 drops of topical proparacaine hydrochloride (0.5%). Prior to implant or sham treatment, eyes received one drop of 10% phenylephrine and 1% tropicamide. 5% betadine solution was used to clean the eye and surrounding area. Group 1 underwent a sham injection procedure, while Group 2 and Group 3 were administered 2× and 3× 30 G × 6 mm Dex-TEG-Dex implants via intravitreal injection, respectively. Animals received buprenorphine 90.02 mg/kg, subcutaneous) for post-procedure analgesia. IOP measurements were performed on both eyes at baseline and designated timepoints up to 52 weeks thereafter using a pneumotonometer. Eyes received 1–2 drops of topical proparacaine hydrochloride (0.5%) prior to IOP measurements, and measurements were taken at approximately the same time of day (±2 h). Wide angle infrared (IR) imaging was performed at baseline and at designated timepoints using a Heidelberg Spectralis® imaging platform. Prior to imaging, eyes were dilated with 1 drop each of 10% phenylephrine and 1% tropicamide. At 3 months and 12 months, a subset of animals from each group was euthanized and both eyes were collected for histopathological analysis. Animals were euthanized via pentobarbital overdose (150 mg/kg, intravenous).

*Histopathology*. Eyes were harvested immediately following euthanasia, placed in Davidson’s solution and stored at room temperature for 24–48 h. Eyes were then transferred to 70% ethanol. Eyes were embedded in paraffin and sectioned using a microtome. Five sections were generated from each eye and stained with hematoxylin and eosin (H&E). All stained sections were microscopically evaluated by an ACVP veterinary pathologist.

### Statistics and reproducibility

All line and bar graph data are expressed as the mean ± standard deviation. Sample sizes were chosen based on previous literature. The number of samples for each experimental group is indicated in each figure legend. Images shown are representative of results across a minimum of three independent samples. Animals were randomized to different treatment groups and all animals were included in analysis. Investigators were not blinded to experiments as part of the analysis required visual observation of test articles that would reveal distinguishing features. Data were analyzed for statistical significance by one-way analysis of variance or unpaired, two-tailed *t-*test where appropriate using Microsoft Excel.

### Ethical compliance

We confirm we have complied with all relevant ethical regulations for animal testing and research, as detailed in the methods. Studies performed at McMaster University were approved by the AREB at McMaster University (AUP# 15-03-09). Protocols performed at Absorption Systems were reviewed and approved by Absorption Systems’ IACUC prior to the initiation of procedures. Protocols performed at the University of Toronto were approved by the Health Sciences Research Ethics Board (REB).

### Reporting summary

Further information on research design is available in the Nature Research Reporting Summary linked to this article.

## Supplementary information

Supplementary Information

Reporting Summary

## Data Availability

The datasets generated during the current study are included in the Supplementary Information files. Data are available from the corresponding author upon reasonable request. Source data are provided with this paper.

## Source data

Source Data

## Code availability

Code is available in the Supplementary Information files.
