# Supplementary material for: Polymer-free corticosteroid dimer implants for controlled and sustained drug delivery
Source: Nat Commun. 2021 May 17;12:2875. doi: 10.1038/s41467-021-23232-7 (PMC8129133; doi:10.1038/s41467-021-23232-7)
Supplement: Supplementary file 1 — Supplementary Information [file 41467_2021_23232_MOESM1_ESM.pdf]

Supplementary Information

Polymer-free corticosteroid dimer implants for controlled and sustained drug delivery

Kyle Battiston[1,#], Ian Parrag[1,#], Matthew Statham[1], Dimitra Louka[1], Hans Fischer[1], Gillian Mackey[1], Adam Daley[1], Fan Gu[1], Emily Baldwin[1], Bingqing Yang[1], Ben Muirhead[2], Emily Anne Hicks[3], Heather Sheardown[2,3], Leonid Kalachev[4], Christopher Crean[5], Jeffrey Edelman[1], J. Paul Santerre[1,6,7,8], Wendy Naimark[1]

1. Ripple Therapeutics, Toronto, Ontario, Canada. 2. School of Biomedical Engineering, McMaster University, Hamilton, Ontario, Canada. 3. Department of Chemical Engineering, McMaster University, Hamilton, Ontario, Canada. 4. Department of Mathematical Sciences, University of Montana, Missoula, Montana, USA. 5. Xyzagen Inc., Pittsboro, North Carolina, USA. 6. Faculty of Dentistry, University of Toronto, Toronto, Ontario, Canada. 7. Institute of Biomaterials and Biomedical Engineering, University of Toronto, Toronto, Ontario, Canada. 8. Translational Biology and Engineering Program, Ted Rogers Centre for Heart Research, Toronto, Ontario, Canada. # These authors contributed equally: Kyle Battiston, Ian Parrag.

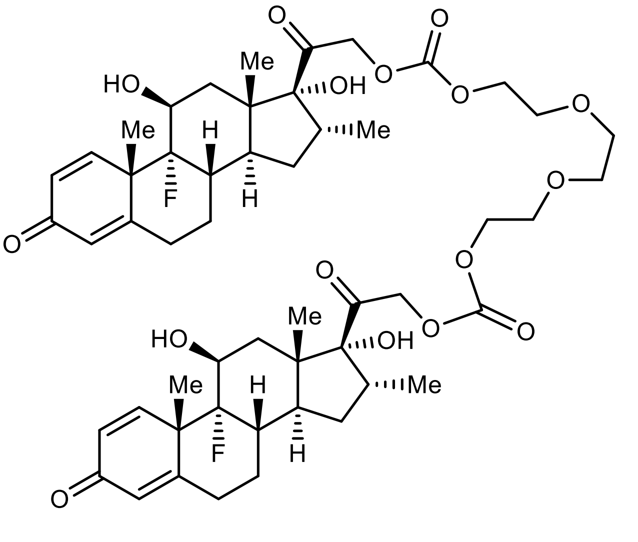

Supplementary Figure 1 Chemical structure of Dex-TEG-Dex (Dex – dexamethasone, TEG – triethylene glycol). ^{1}H NMR (400 MHz, DMSO-d6) δ 7.29 (d, J = 10.1 Hz, 2H), 6.22 (dd, J = 10.1, 1.9 Hz, 2H), 6.01 (t, J = 1.7 Hz, 2H), 5.40 (dd, J = 4.9, 1.4 Hz, 2H), 5.18 (s, 2H), 5.09 (d, J = 17.8 Hz, 2H), 4.79 (d, J = 17.7 Hz, 2H), 4.25 – 4.19 (m, 4H), 4.19 – 4.10 (m, 2H), 3.68 – 3.60 (m, 4H), 3.57 (s, 4H), 2.89 (ddd, J = 11.2, 7.3, 4.1 Hz, 2H), 2.62 (qd, J = 12.0, 5.5 Hz, 2H), 2.45 – 2.32 (m, 2H), 2.30 (dd, J = 7.1, 3.5 Hz, 2H), 2.22 – 2.05 (m, 4H), 1.81 – 1.73 (m, 2H), 1.64 (q, J = 11.7 Hz, 2H), 1.54 (dd, J = 13.8, 2.0 Hz, 2H), 1.49 (s, 6H), 1.35 (qd, J = 12.8, 5.1 Hz, 2H), 1.07 (ddd, J = 12.0, 8.0, 4.0 Hz, 2H), 0.90 (s, 6H), 0.80 (d, J = 7.2 Hz, 6H); ^{13}C NMR (100 MHz, DMSO-d6) δ 204.9, 185.3, 167.0, 154.4, 152.7, 129.0, 124.1, 105.9, 102.1, 100.4, 90.4, 70.7, 70.3, 69.7, 68.1, 67.2, 48.0, 47.8, 43.3, 35.7, 35.4, 33.7, 33.5, 31.9, 30.3, 27.3, 23.0, 22.9, 16.2, 15.1; HRMS (m/z): $[M]^+$ calcd. for $C_{52}H_{69}F_2O_{16}$, 987.4554; found, 987.4549.

(a)

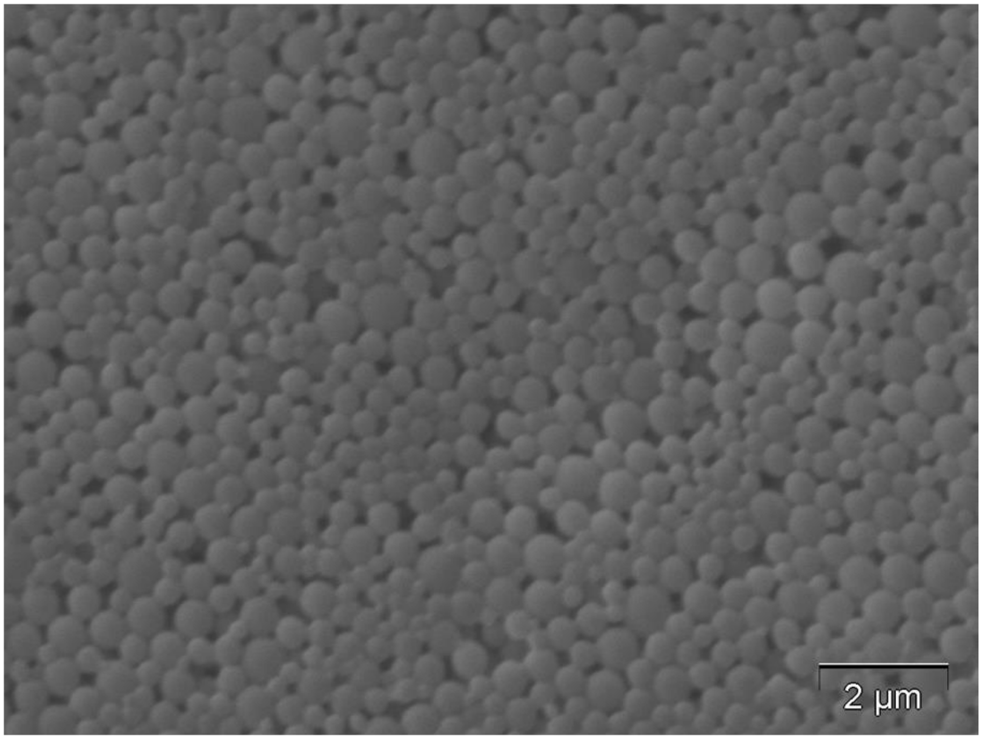

(b)

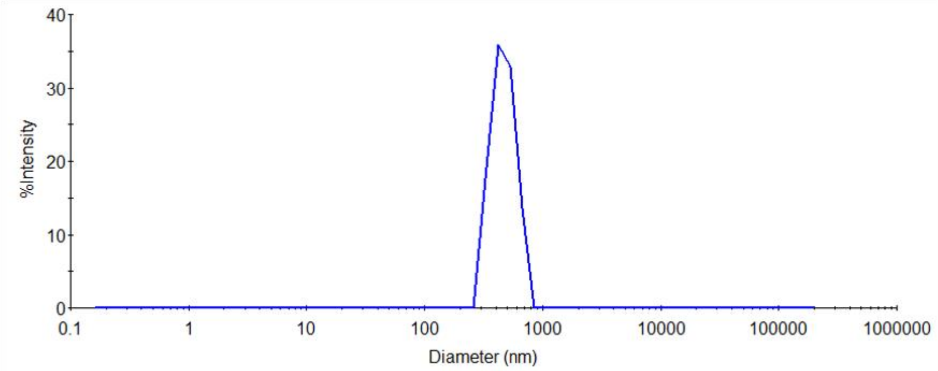

Supplementary Figure 2 (a) Scanning electron micrograph showing nanoparticles fabricated from TA-TEG-TA. (b) Dynamic light scattering assessment of nanoparticle size distribution, indicating a mean particle diameter of 448 nm and a polydispersity index of 0.07.

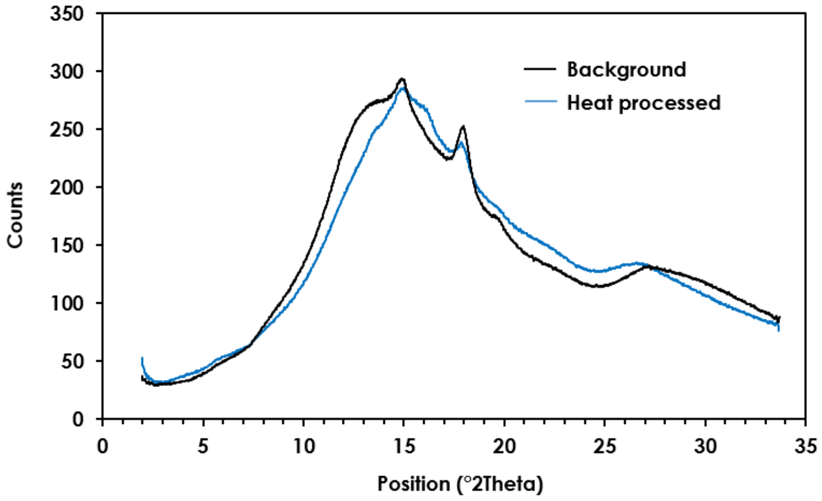

Supplementary Figure 3 Powder X-ray diffraction (PXRD) diffractogram of heat-processed Dex-TEG-Dex (Dex – dexamethasone, TEG – triethylene glycol) compared to the background signal from the sample holder.

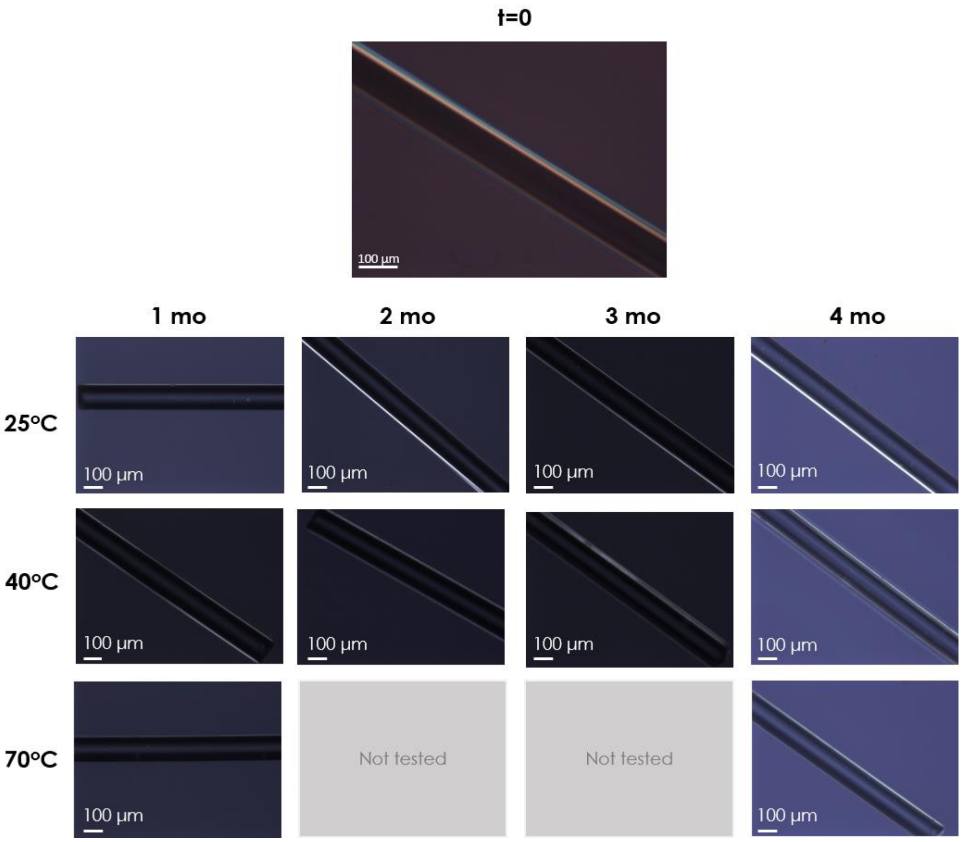

Supplementary Figure 4 Shelf-life stability assessment of Dex-TEG-Dex extruded rods. PLM images indicating an absence of crystallinity in as-made extruded rods as well as following the indicated shelf-life storage conditions.

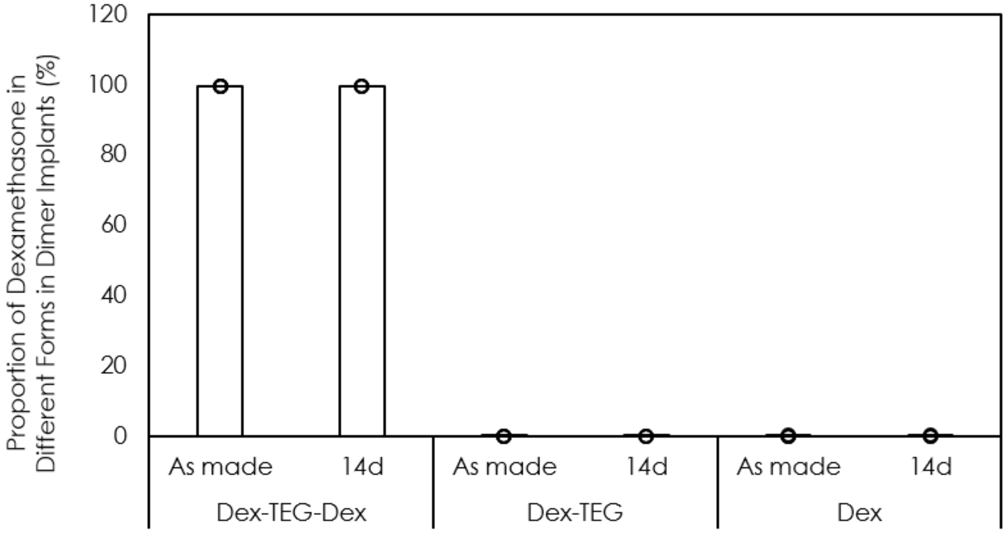

Supplementary Figure 5 Proportion of dexamethasone in different forms, namely Dex-TEG-Dex, Dex-TEG, and free Dex (Dex – dexamethasone, TEG – triethylene glycol) in heat-molded pellets as-made and following release in 100% fetal bovine serum (FBS), demonstrating that the bulk of the material remains the dimer following submersion in release buffer for 14 days. N=4 samples for as made and N=3 samples for 14 days. Data represent the mean ± standard deviation.

(a) (b)

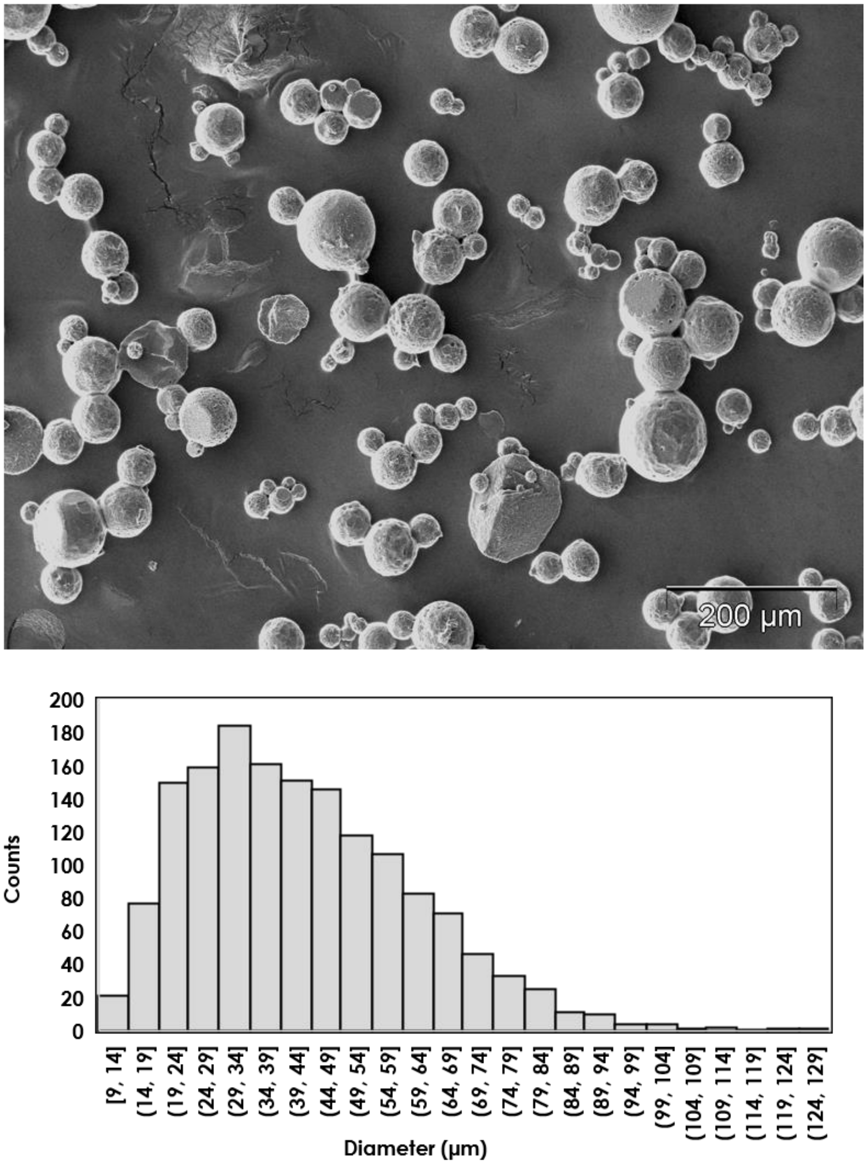

Supplementary Figure 6 (a) Scanning electron micrograph showing microparticles formed from TA-TEG-TA. (b) Semi-quantitative assessment of microparticle size distribution from SEM images from three batches of microparticles (diameter = 42.2 ± 18.2 µm).

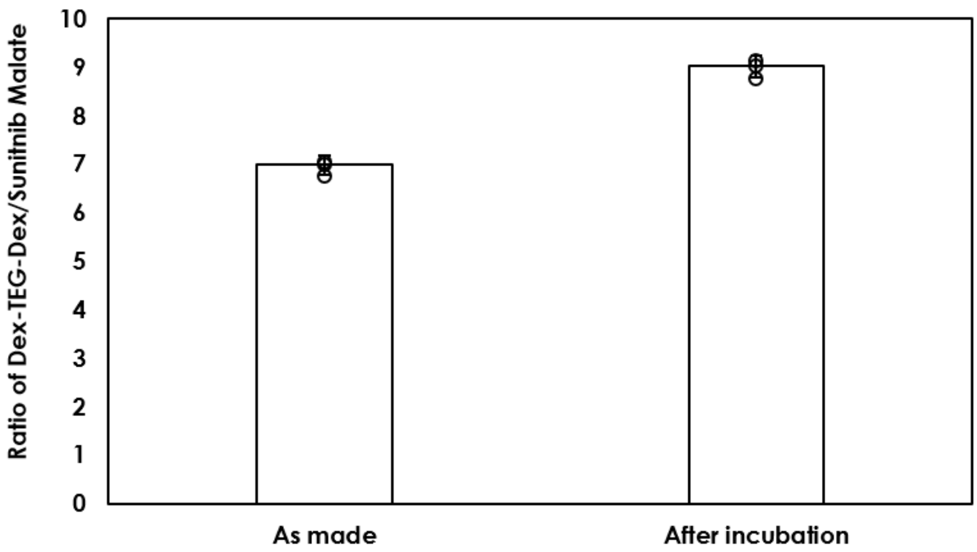

Supplementary Figure 7 Assessment of the ratio of drug dimer to free drug (sunitinib malate) as-made and following release in phosphate buffered saline (PBS). N=3 samples. Data represent the mean ± standard deviation.

.

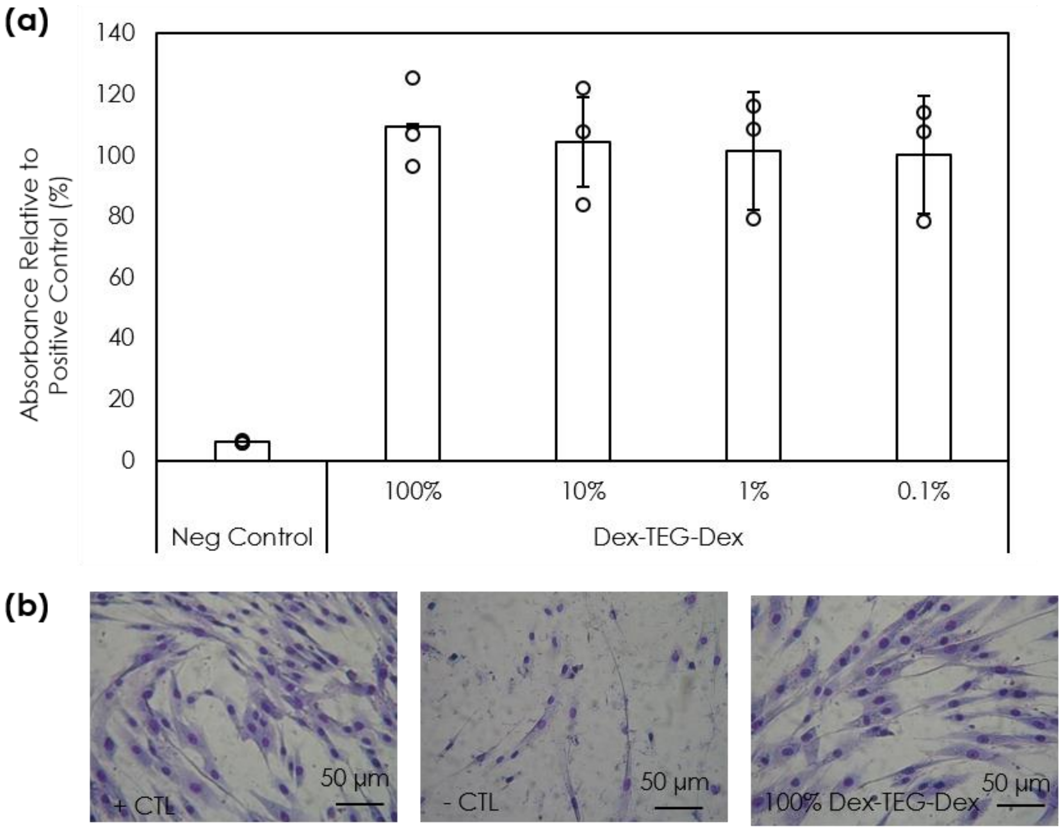

Supplementary Figure 8 (a) Metabolic activity of human dermal fibroblasts exposed to maximum soluble levels of Dex-TEG-Dex (Dex – dexamethasone, TEG – triethylene glycol) (100%) and dilutions of 10%, 1%, and 0.1%, compared to negative control (5% dimethyl sulfoxide in culture medium). N=3. Data are the mean ± standard deviation. Measurements are normalized to the positive control, which represents 100%. (b) Light microscope images of human dermal fibroblasts following exposure Dex-TEG-Dex, and negative and positive control conditions and exposure to Diff-Quik staining (blue – nuclei, purple – cytoplasm).

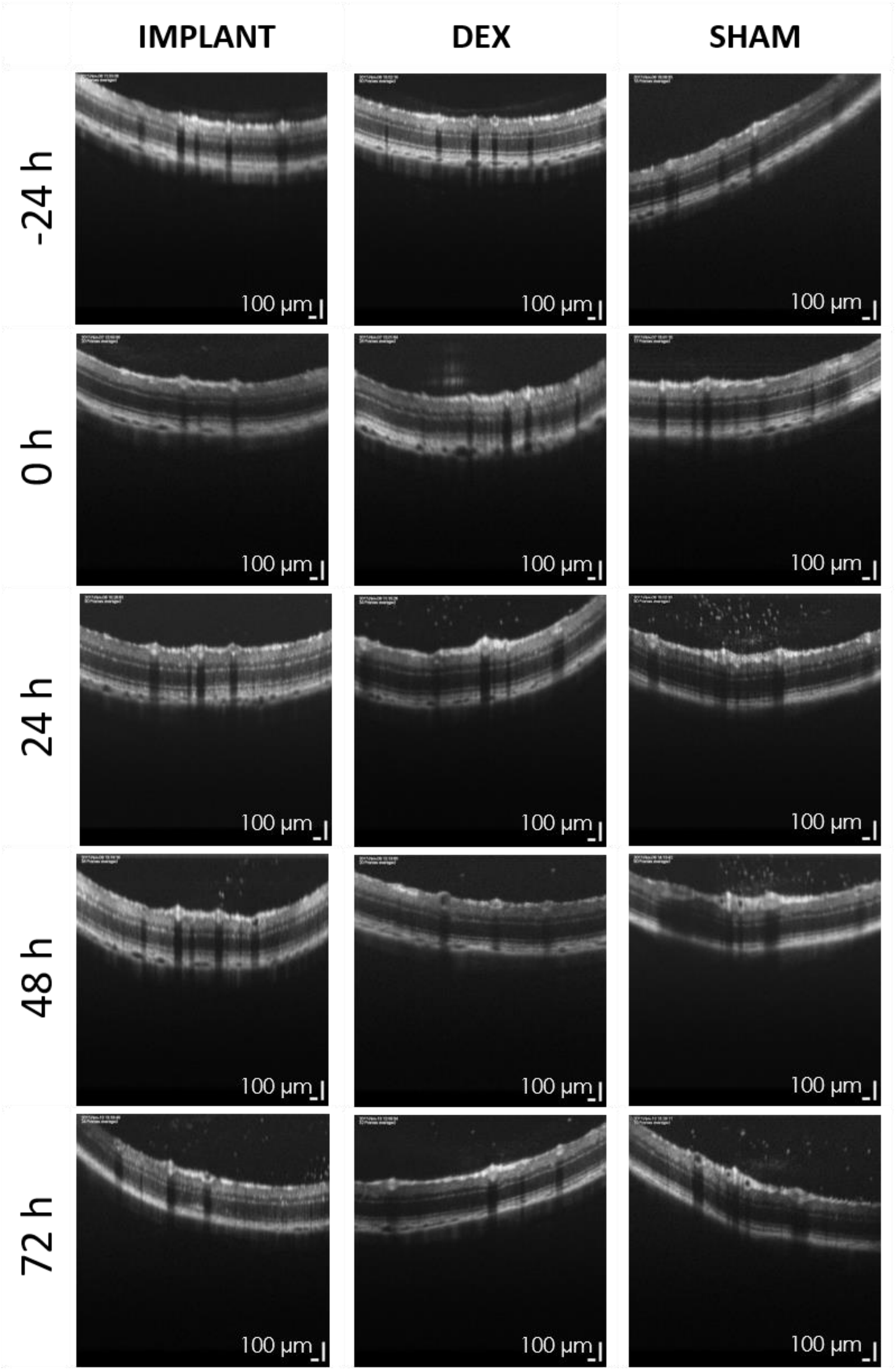

Supplementary Figure 9 Representative optical coherence tomography (OCT) images for eyes receiving a sham injection, dexamethasone (Dex) eye drops, or a Dex-TEG-Dex (TEG – triethylene glycol) implant at t = -24 h, 0 h, 24 h, 48, and 72 h with respect to lipopolysaccharide (LPS) injection.

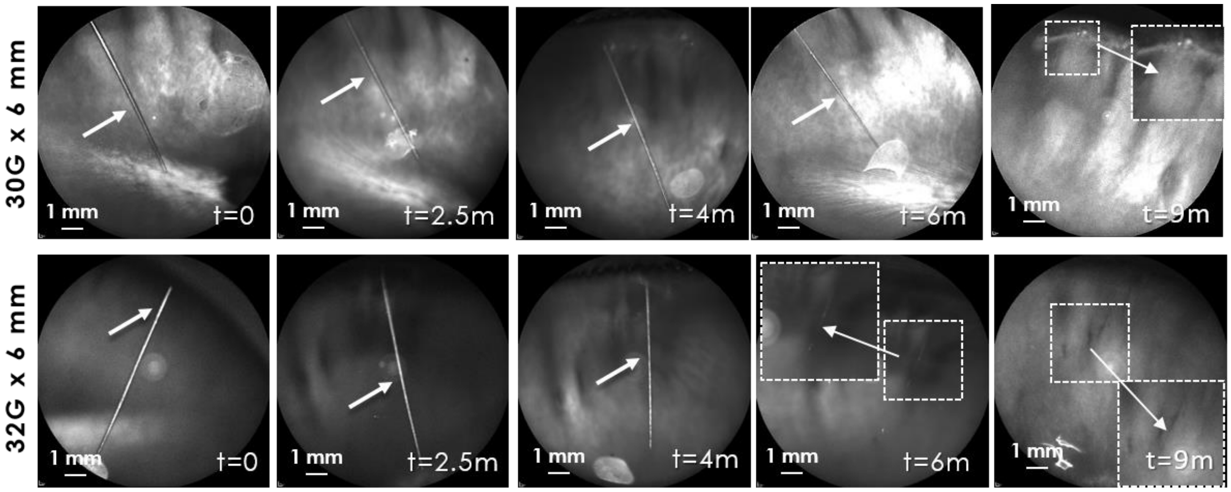

Supplementary Figure 10 Representative IR images of 30G x 6 mm and 32G x 6 mm Dex-TEG-Dex (Dex – dexamethasone, TEG – triethylene glycol) in the intravitreal space of the rabbit eye.

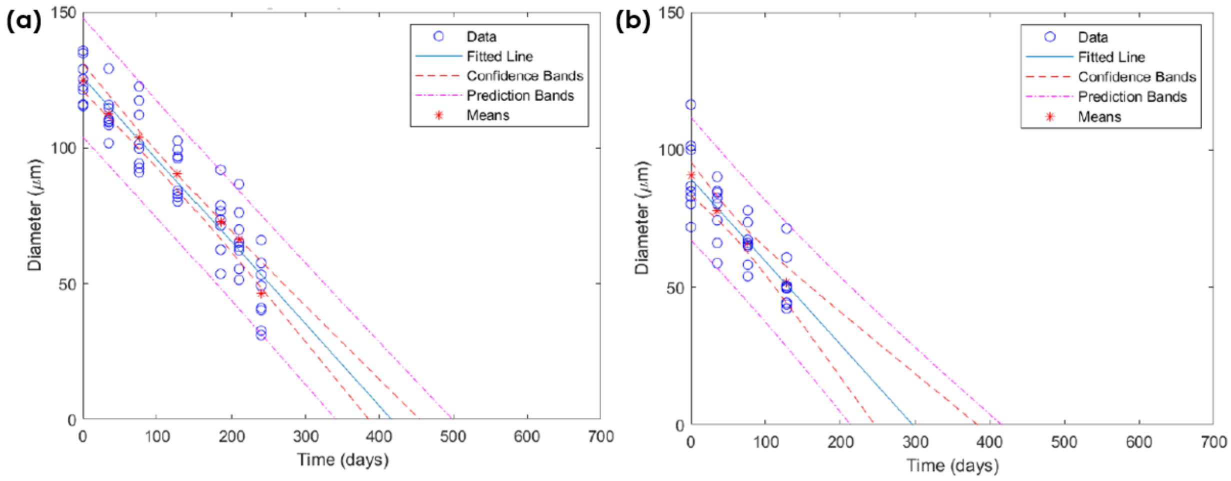

Supplementary Figure 11 Modeling[1,2] of implant diameter following intravitreal administration of Dex-TEG-Dex (Dex – dexamethasone, TEG – triethylene glycol) implants in the rabbit eye for (a) 30G x 6 mm and (b) 32G x 6 mm implants.

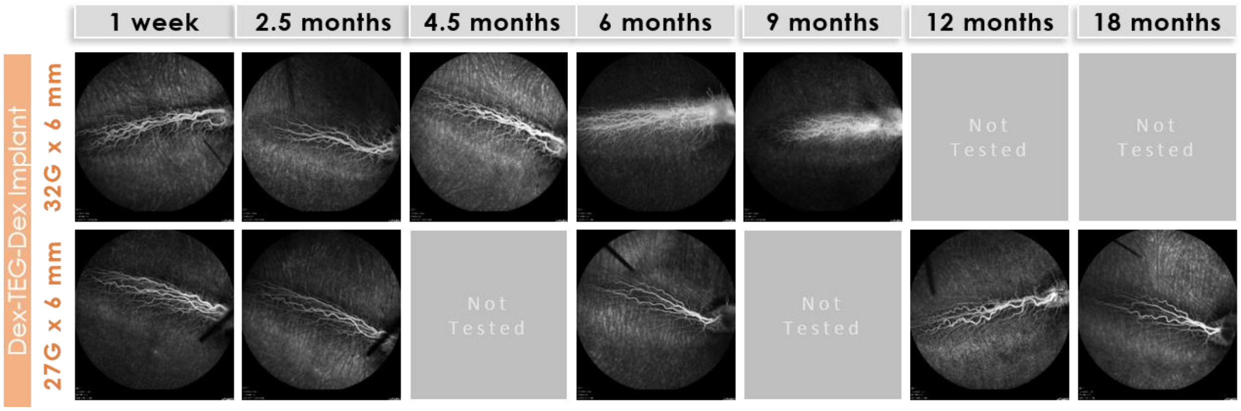

Supplementary Figure 12 Fluorescein angiograms of the back of the eye following intravitreal injection of vascular endothelial growth factor (VEGF) for eyes receiving 32G x 6 mm or 27G x 6 mm dexamethasone dimer (Dex-TEG-Dex, Dex – dexamethasone, TEG – triethylene glycol) implants at indicated time points for up to 18 months.

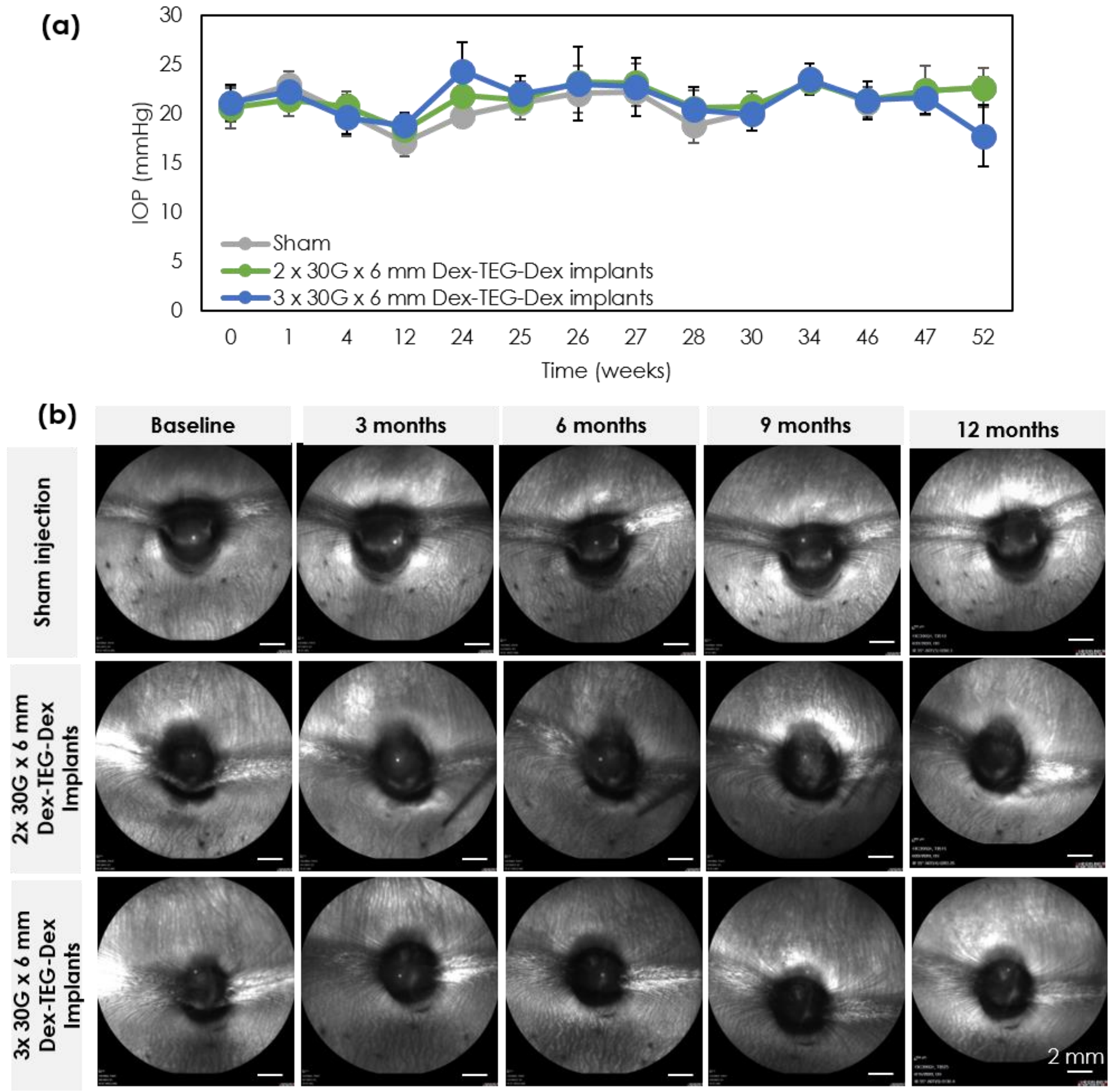

Supplementary Figure 13 Safety assessments for intravitreal Dex-TEG-Dex implants. (a) Intraocular pressure measurements (Data are the mean ± S.D. N=12 eyes up to week 12, N=6 eyes thereafter.) and (b) IR fundus images following sham injections and intravitreal implantation of 2x and 3x 30G x 6 mm Dex-TEG-Dex implants for up to 12 months.

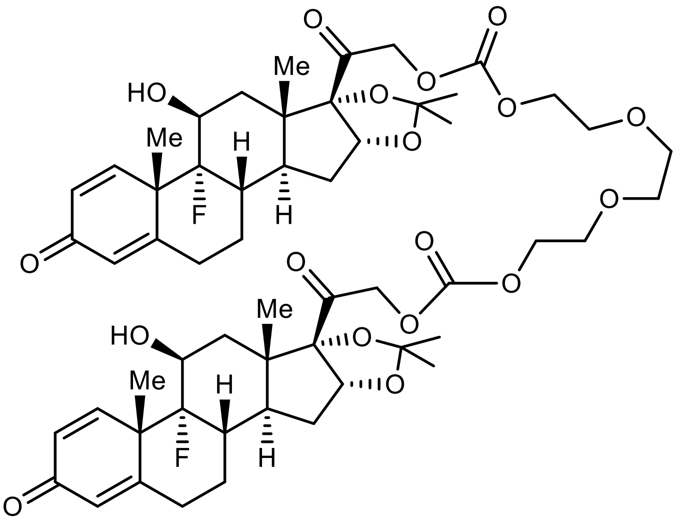

Supplementary Figure 14 Chemical structure of TA-TEG-TA (TA – triamcinolone acetonide, TEG – triethylene glycol). ^{1}H NMR (400 MHz, DMSO-d6) δ 7.29 (d, J = 10.1 Hz, 2H), 6.23 (dd, J = 10.1, 1.9 Hz, 2H), 6.02 (t, J = 1.6 Hz, 2H), 5.48 (dd, J = 4.9, 1.5 Hz, 2H), 5.24 – 5.13 (m, 2H), 4.87 (d, J = 4.7 Hz, 2H), 4.82 – 4.69 (m, 2H), 4.28 – 4.22 (m, 4H), 4.20 (d, J = 2.8 Hz, 2H), 3.69 – 3.60 (m, 4H), 3.57 (s, 4H), 2.62 (dt, J = 13.2, 6.8 Hz, 2H), 2.44 (td, J = 12.1, 5.2 Hz, 2H), 2.33 (dd, J = 13.5, 4.3 Hz, 2H), 2.03 (d, J = 13.5 Hz, 2H), 1.93 (td, J = 12.2, 6.6 Hz, 2H), 1.86 – 1.78 (m, 2H), 1.69 (d, J = 13.5 Hz, 2H), 1.64 – 1.51 (m, 4H), 1.49 (s, 6H), 1.35 (m, 8H), 1.14 (s, 6H), 0.83 (s, 6H); ^{13}C NMR (100 MHz, DMSO-d6) δ 203.5, 185.2, 166.52, 166.50, 154.2, 152.4, 129.0, 124.3, 111.0, 101.8, 100.1, 97.0, 81.1, 70.4, 70.0, 69.7, 68.1, 67.4, 47.8, 47.6, 45.2, 42.8, 35.9, 33.0, 32.5, 32.3, 30.1, 27.5, 26.3, 25.3, 22.82, 22.77, 16.0; HRMS (m/z): $[M]^+$ calcd. for $C_{56}H_{73}F_2O_{18}$, 1071.4765; found, 1071.4763.

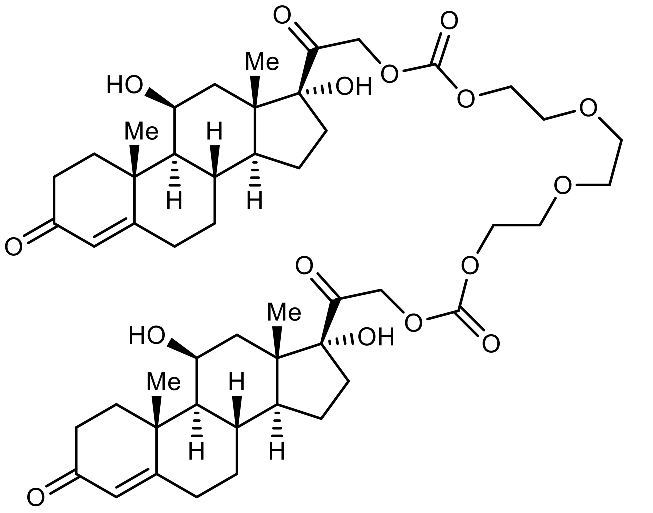

Supplementary Figure 15 Chemical structure of HC-TEG-HC (HC – hydrocortisone, TEG – triethylene glycol). ^{1}H NMR (400 MHz, DMSO-d6) δ 5.59 – 5.54 (m, 2H), 5.43 (s, 2H), 5.11 (d, J = 17.7 Hz, 2H), 4.77 (d, J = 17.6 Hz, 2H), 4.36 (d, J = 4.0 Hz, 2H), 4.26 (p, J = 3.5 Hz, 2H), 4.24 – 4.17 (m, 4H), 3.68 – 3.61 (m, 4H), 3.57 (s, 4H), 2.49 – 2.30 (m, 6H), 2.24 – 2.14 (m, 4H), 2.10 (dt, J = 11.4, 3.7 Hz, 2H), 1.96 – 1.88 (m, 6H),

1.78 (td, J = 13.4, 4.4 Hz, 4H), 1.70 – 1.58 (m, 6H), 1.46 (dt, J = 14.4, 7.9 Hz, 2H), 1.36 (s, 6H), 1.08 – 0.92 (m, 2H), 0.87 (dd, J = 11.1, 3.2 Hz, 2H), 0.78 (s, 6H); ^{13}C NMR (100 MHz, DMSO-d6) δ 205.3, 198.0, 172.3, 154.4, 121.5, 88.6, 70.3, 69.7, 68.1, 67.1, 66.3, 55.5, 51.6, 46.9, 38.9, 38.8, 34.1, 33.5, 33.1, 32.8, 31.4, 31.1, 23.3, 20.5, 16.6; HRMS (m/z): $[M]^+$ calcd. for $C_{50}H_{70}NaO_{16}$, 949.4556; found, 949.4565.

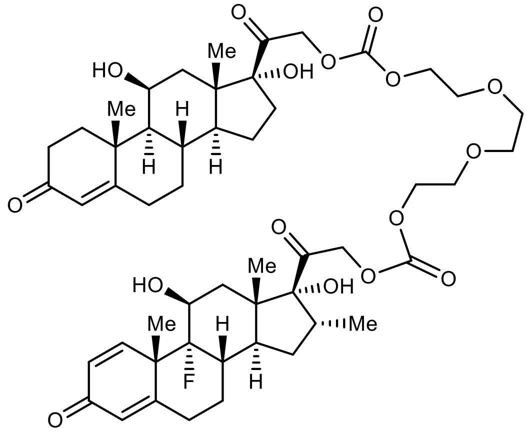

Supplementary Figure 16 Chemical structure of HC-TEG-Dex (HC – hydrocortisone, TEG – triethylene glycol, Dex – dexamethasone). ^{1}H NMR (400 MHz, DMSO-d6) δ 7.29 (d, J = 10.1 Hz, 1H), 6.22 (dd, J = 10.1, 1.9 Hz, 1H), 6.00 (t, J = 1.7 Hz, 1H), 5.55 (d, J = 1.4 Hz, 1H), 5.42 (s, 1H), 5.39 (dd, J = 4.9, 1.4 Hz, 1H), 5.17 (s, 1H), 5.10 (dd, J = 17.7, 11.0 Hz, 2H), 4.78 (dd, J = 17.7, 6.2 Hz, 2H), 4.34 (d, J = 3.9 Hz, 1H), 4.27 (p, J = 3.4 Hz, 1H), 4.24 – 4.19 (m, 4H), 4.16 (q, J = 5.4 Hz, 1H), 3.68 – 3.61 (m, 4H), 3.57 (s, 4H), 2.89 (ddd, J = 11.2, 7.3, 4.1 Hz, 1H), 2.60 (dt, J = 13.4, 6.9 Hz, 1H), 2.55 – 2.45 (m, 2H), 2.45 – 2.26 (m, 4H), 2.25 – 2.04 (m, 5H), 1.96 – 1.84 (m, 3H), 1.84 – 1.72 (m, 2H), 1.72 – 1.58 (m, 4H), 1.58 – 1.51 (m, 1H), 1.49 (s, 3H), 1.36 (s, 4H), 1.33 – 1.21 (m, 2H), 1.13 – 1.03 (m, 1H), 1.03 – 0.93 (m, 1H), 0.90 (s, 3H), 0.88 – 0.83 (m, 1H), 0.83 – 0.74 (m, 6H); ^{13}C NMR (100 MHz, DMSO-d6) δ 205.3, 204.9, 198.1, 185.3, 172.3, 167.0, 154.4, 152.7, 129.0, 124.2, 121.5, 102.1, 100.4, 90.5, 88.6, 70.7, 70.3, 69.8, 68.2, 67.2, 66.4, 59.8, 55.6, 54.9, 51.6, 48.08, 48.07, 47.9, 47.0, 43.3, 39.0, 38.9, 36.3, 35.7, 35.5, 34.1, 33.7, 33.53, 33.49, 33.2, 32.8, 31.9, 31.4, 31.2, 30.3, 27.3, 24.3, 23.7, 23.3, 23.02, 22.96, 20.8, 20.5, 16.6, 16.3, 15.1, 14.1; HRMS (m/z): $[M]^+$ calcd. for $C_{51}H_{69}FNaO_{16}$, 979.4462; found, 979.4458.

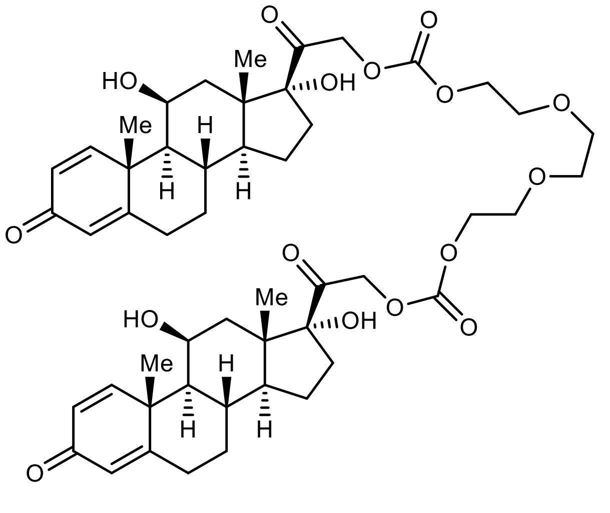

Supplementary Figure 17 Chemical structure of Pred-TEG-Pred (Pred – prednisolone, TEG – triethylene glycol). ^{1}H NMR (400 MHz, DMSO-d6) δ 7.32 (d, J = 10.1 Hz, 2H), 6.16 (dd, J = 10.1, 1.9 Hz, 2H), 5.91 (t, J = 1.5 Hz, 2H), 5.42 (s, 2H), 5.10 (d, J = 17.7 Hz, 2H), 4.76 (d, J = 17.7 Hz, 2H), 4.72 (d, J = 3.9 Hz, 2H), 4.32 – 4.25 (m, 2H), 4.25 – 4.18 (m, 4H), 3.68 – 3.60 (m, 4H), 3.57 (s, 6H), 2.52 (m, 4H), 2.29 (dd, J = 12.9, 4.2 Hz, 2H), 1.89 (dd, J = 13.6, 3.6 Hz, 2H), 1.70 – 1.57 (m, 6H), 1.45 (dt, J = 14.4, 7.5 Hz, 4H), 1.39 (s, 6H), 1.31 (dd, J = 11.2, 5.9 Hz, 2H), 0.99 (td, J = 13.0, 4.6 Hz, 2H), 0.89 (dd, J = 11.0, 3.4 Hz, 2H), 0.80 (s, 6H); ^{13}C NMR (100 MHz, DMSO-d6) δ 205.3, 185.1, 170.4, 156.6, 154.4, 127.1, 121.6, 88.5, 70.3, 69.7, 68.3, 68.1, 67.1, 55.3, 51.0, 47.1, 43.7, 38.7, 34.0, 33.1, 31.3, 30.9, 23.5, 20.9, 16.5; HRMS (m/z): $[M]^+$ calcd. for $C_{50}H_{66}NaO_{16}$, 945.4243; found, 945.4248.

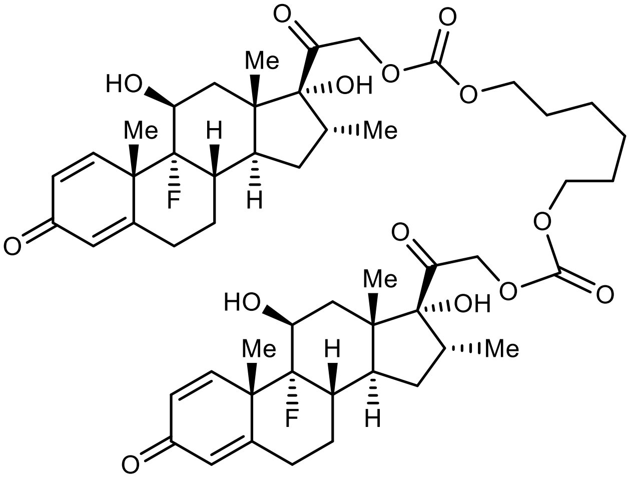

Supplementary Figure 18 Chemical structure of Dex-Hex-Dex (Dex – dexamethasone, Hex – 1,6-hexanediol). ^{1}H NMR (400 MHz, DMSO-d6) δ 7.29 (d, J = 10.2 Hz, 2H), 6.22 (dd, J = 10.1, 1.9 Hz, 2H), 6.01 (t, J = 1.6 Hz, 2H), 5.40 (dd, J = 4.9, 1.4 Hz, 2H), 5.17 (s, 2H), 5.07 (d, J = 17.7 Hz, 2H), 4.78 (d, J = 17.7 Hz, 2H), 4.11 (q, J = 6.3 Hz, 6H), 2.89 (ddd, J = 11.2, 7.3, 4.1 Hz, 2H), 2.68 – 2.55 (m, 2H), 2.45 – 2.33 (m, 2H), 2.33 – 2.26 (m, 2H), 2.21 – 2.06 (m, 4H), 1.81 – 1.73 (m, 2H), 1.71 – 1.59 (m, 6H), 1.59 – 1.51 (m, 2H), 1.49 (s, 6H), 1.43 – 1.29 (m, 6H), 1.07 (ddd, J = 12.0, 8.0, 4.1 Hz, 2H), 0.90 (s, 6H), 0.79 (d, J = 7.2 Hz, 6H);

^{13}C NMR (100 MHz, DMSO-d6) δ 205.0, 185.3, 167.03, 167.01, 154.4, 152.7, 129.0, 124.1, 102.1, 100.4, 90.4, 70.7, 70.6, 70.3, 67.7, 48.1, 48.0, 47.8, 43.3, 35.7, 35.5, 33.7, 33.5, 31.9, 30.3, 28.0, 27.3, 24.7, 23.0, 22.9, 16.2, 15.1; HRMS (m/z): $[M]^+$ calcd. for $C_{52}H_{68}F_2NaO_{14}$, 977.4469; found, 977.4471.

Supplementary References

1. Haario, H. & Kalachev, L. Asymptotic analysis of a complex reaction scheme in solid-liquid system. *Chem. Eng. Sci.* **58**, 2823–2834 (2003).
2. Smith, B. T. *Remington Education: Physical Pharmacy*. (Pharmaceutical Press, 2015).
