# Supplementary material for: Polymer-free corticosteroid dimer implants for controlled and sustained drug delivery
Source: Nat Commun. 2021 May 17;12:2875. doi: 10.1038/s41467-021-23232-7 (PMC8129133; doi:10.1038/s41467-021-23232-7)
Supplement: Supplementary file 2 — Reporting Summary [file 41467_2021_23232_MOESM2_ESM.pdf]

nature research

Corresponding author(s): Wendy Naimark

Last updated by author(s): Mar 10, 2021

Reporting Summary

Nature Research wishes to improve the reproducibility of the work that we publish. This form provides structure for consistency and transparency in reporting. For further information on Nature Research policies, see our Editorial Policies and the Editorial Policy Checklist.

Statistics

For all statistical analyses, confirm that the following items are present in the figure legend, table legend, main text, or Methods section.

n/a	Confirmed	
☐	☒	The exact sample size (*n*) for each experimental group/condition, given as a discrete number and unit of measurement
☐	☒	A statement on whether measurements were taken from distinct samples or whether the same sample was measured repeatedly
☐	☒	The statistical test(s) used AND whether they are one- or two-sided<br>*Only common tests should be described solely by name; describe more complex techniques in the Methods section.*
☒	☐	A description of all covariates tested
☒	☐	A description of any assumptions or corrections, such as tests of normality and adjustment for multiple comparisons
☐	☒	A full description of the statistical parameters including central tendency (e.g. means) or other basic estimates (e.g. regression coefficient) AND variation (e.g. standard deviation) or associated estimates of uncertainty (e.g. confidence intervals)
☐	☒	For null hypothesis testing, the test statistic (e.g. *F*, *t*, *r*) with confidence intervals, effect sizes, degrees of freedom and *P* value noted<br>*Give P values as exact values whenever suitable.*
☒	☐	For Bayesian analysis, information on the choice of priors and Markov chain Monte Carlo settings
☒	☐	For hierarchical and complex designs, identification of the appropriate level for tests and full reporting of outcomes
☒	☐	Estimates of effect sizes (e.g. Cohen's *d*, Pearson's *r*), indicating how they were calculated

Our web collection on statistics for biologists contains articles on many of the points above.

Software and code

Policy information about availability of computer code

Data collection	All commercial software suites are specified. 1) LAS EZ v.3.2.0 software (Leica). 2) Diffrac.Eva software version 4.2.1 (Bruker). 3) Agilent OpenLAB ChemStation Rev. C.01.07. 4) ZoomBrowser EX software (Canon, version 5.0.0.142). 5) Microsoft Excel 2016. 6) ImageJ v.1.8.0. 7) Leica Application Suite X Version 3.0.12.21488, 8) MathWorks MATLAB R2020b.
Data analysis	All commercial software suites are specified.

For manuscripts utilizing custom algorithms or software that are central to the research but not yet described in published literature, software must be made available to editors and reviewers. We strongly encourage code deposition in a community repository (e.g. GitHub). See the Nature Research guidelines for submitting code & software for further information.

Data

Policy information about availability of data

All manuscripts must include a data availability statement. This statement should provide the following information, where applicable:

- Accession codes, unique identifiers, or web links for publicly available datasets
- A list of figures that have associated raw data
- A description of any restrictions on data availability

The datasets generated during the current study are included in the Supplementary Information files. In the source data file, the data can be found for Figs. 2c, 4b-h, 5a-h, 6a-c, 7d-e, 8b-e, 9b and Supplementary Figs. 5, 7, 8, and 13.

Field-specific reporting

Please select the one below that is the best fit for your research. If you are not sure, read the appropriate sections before making your selection.

☒ Life sciences ☐ Behavioural & social sciences ☐ Ecological, evolutionary & environmental sciences

For a reference copy of the document with all sections, see nature.com/documents/nr-reporting-summary-flat.pdf

Life sciences study design

All studies must disclose on these points even when the disclosure is negative.

Sample size	Sample size calculations were not performed. Sample sizes were chosen based on previous literature for similar types of studies, such as in vitro drug release studies and in vivo studies (e.g. Farah et al. Nat Mater 2019;18(8):892-904). The number of samples for each experimental group is indicated in each figure legend.
Data exclusions	A single data point from Fig. 8d was excluded as an outlier based on statistical analysis.
Replication	All attempts at replication were successful. The number of samples for each experimental group is indicated in each figure legend.
Randomization	Animals were randomized to different treatment groups and all animals were included in analysis. For other studies, after samples were prepared they were randomly allocated to different experimental groups.
Blinding	Investigators were not blinded to experiments. Data collection and analysis required visual observation and inspection of the test articles that would reveal distinguishing features.

Reporting for specific materials, systems and methods

We require information from authors about some types of materials, experimental systems and methods used in many studies. Here, indicate whether each material, system or method listed is relevant to your study. If you are not sure if a list item applies to your research, read the appropriate section before selecting a response.

Materials & experimental systems

n/a	Involved in the study	
☒	☐	Antibodies
☐	☒	Eukaryotic cell lines
☒	☐	Palaeontology and archaeology
☐	☒	Animals and other organisms
☐	☒	Human research participants
☒	☐	Clinical data
☒	☐	Dual use research of concern

Methods

n/a	Involved in the study	
☒	☐	ChIP-seq
☒	☐	Flow cytometry
☒	☐	MRI-based neuroimaging

Eukaryotic cell lines

Policy information about cell lines

Cell line source(s)	Cell Applications
Authentication	None of the cell lines used were authenticated other than procedures undertaken by the vendor the cells were sourced from.
Mycoplasma contamination	The cell lines were not tested for mycoplasma contamination.
Commonly misidentified lines (See ICLAC register)	No commonly misidentified cell lines were used in this study.

Animals and other organisms

Policy information about studies involving animals; ARRIVE guidelines recommended for reporting animal research

Laboratory animals	Animals used and their details are reported in the methods section. Briefly, Dutch-belted rabbits (male, 1.84-2.49 kg, 5-6 months old), New Zealand white rabbits (female, 1.5-2.5 kg, age commensurate with weight), New Zealand white rabbits (female, 2.73-3.83 kg, 3-4 months old), and Brown Norway rats (female, 3-5 weeks old, 80-120 g)
Wild animals	No wild animals were used in the studies.

Field-collected samples	No samples collected from the field were used in the studies.
Ethics oversight	Ethics oversight was provided by the ethics review boards of the institutions where the studies were conducted, including the Animal Research Ethics Board at McMaster University, the Health Sciences Research Ethics Board at the University of Toronto, and Absorption Systems' Institutional Animal Care and Use Committee.

Note that full information on the approval of the study protocol must also be provided in the manuscript.

Human research participants

Policy information about studies involving human research participants

Population characteristics	Participants were healthy volunteers and were not screened for age, gender, or sex.
Recruitment	Participants were recruited from volunteers within Ripple Therapeutics and the research institute of the co-authors.
Ethics oversight	Health Sciences Research Ethics Board at the University of Toronto

Note that full information on the approval of the study protocol must also be provided in the manuscript.
